# Optimizing single-session CBT delivery in an 8-session longitudinal therapeutic assessment (FRAX-TA) for women with *FMR1* Premutation

**DOI:** 10.3389/fnmol.2026.1718675

**Published:** 2026-04-24

**Authors:** Federica Alice Maria Montanaro, Giuseppina Spano, Randi J. Hagerman, Giancarlo Logroscino, Andrea Bosco

**Affiliations:** 1 Department of Education, Psychology, Communication, University of Bari Aldo Moro, Bari, Italy; 2 Department of Clinical Research in Neurology, Center for Neurodegenerative Diseases and the Aging Brain, University of Bari Aldo Moro/Pia Fond. Card. G. Panico, Tricase, Italy; 3 Department of Psychology and Health Science, Pegaso University, Naples, Italy; 4 UC Davis MIND Institute, UC Davis Health, Sacramento, CA, United States; 5 Department of Pediatrics, School of Medicine, University of California, Davis, Sacramento, CA, United States

**Keywords:** cognitive behavioral therapy, FMR1 premutation, Fragile X Premutation Associated Conditions, Fragile X Syndrome, FXAND, single-session intervention, therapeutic assessment, Fragile X Disorders

## Abstract

**Background:**

Therapeutic Assessment (TA) is a client-centered approach that uses psychological evaluation to promote therapeutic change. While TA has shown benefits across populations, its application to genetically at-risk groups remains limited. Women with the *FMR1* premutation (PM) are vulnerable to mood, anxiety, and cognitive symptoms, collectively referred to as Fragile X-associated Neuropsychiatric Disorders (FXAND). However, tailored psychological assessments for this population are still lacking. This study introduces FRAX-TA, a TA-based protocol for women with the PM, integrating a Single-Session Cognitive Behavioral Therapy (SS-CBT) component: the Psychoeducational Assessment (PA). The aim was to offer psychological support, while collecting quantitative data. We also explored whether varying the timing of the PA influences outcomes.

**Methods:**

Eighty-one Italian women with genetically confirmed PM (M age = 50.5 ± 9.41) completed an 8-week TA protocol based on the Cognitive Behavioral Assessment for Outcome Evaluation (CBA-VE). Participants were randomized into four groups receiving the PA during the 4th, 6th, 8th, or 10th week from baseline. Mixed-design Bayesian ANOVAs assessed changes across timepoints (baseline, post-PA, follow-up) and the effect of the PA timing on the five CBA-OE psychological domains (anxiety, wellbeing, perception of positive change, depression, and psychological distress). An anonymous feedback questionnaire evaluated participant experiences.

**Results:**

Participants showed significant reductions in the CBA-VE anxiety, depression, and distress scores. The PA had both immediate and delayed effects, particularly for depression and anxiety. Mid-phase delivery led to more stable improvements in CBA-VE perceived positive change. Qualitative feedback indicated high satisfaction and emotional support.

**Conclusion:**

FRAX-TA appears effective for women with the PM, providing therapeutic benefit even without ongoing treatment. Findings underscore the added value of SS-CBT within assessment and suggest that repeated sessions may enhance symptom recognition and prompt further care. Future studies should include control groups, larger samples, and examine personalized timing to optimize outcomes.

## 1 Introduction

Accurate assessment is a cornerstone of effective psychological intervention, yet its role as a therapeutic agent remains debated. Traditionally viewed as a precursor to treatment, assessment is often framed as a process of gathering diagnostic information, formulating case conceptualizations, and informing treatment planning (Durosini and Aschieri, 2021). In both clinical and research contexts, it is typically approached as a procedural step focused on data collection and classification. When professionals engage with clients, the primary objective frequently involve mapping the individual’s functioning onto diagnostic categories, identify symptomatic patterns, and provide treatment recommendations (Groth-Marnat and Wright, 2016). While methodologically rigorous, this model may overlook the potential of assessment as a site of therapeutic engagement. By prioritizing classification, clinicians may risk missing opportunities to foster insight, promote self-understanding, and initiate change. Consequently, reports may be rich in data yet emotionally disengaged, limiting their impact on client wellbeing (Finn, 2005). Moreover, when assessment lacks a therapeutic connection, clients may feel misunderstood, leading them to minimize or withhold symptoms. This, in turn, may cause clinicians to underestimate the complexity or severity of presenting concerns, thereby reducing clinical and research utility (Finn, 2007). To address these limitations, a growing body of work has advocated for collaborative, client-centered models of assessment, such as the Therapeutic Assessment (TA) developed by Stephen Finn and colleagues (Finn and Tonsager, 1992, 1997). Grounded in clinical science and humanistic principles, TA emphasizes co-constructing meaning from assessment results, encouraging curiosity, clarity, and destigmatization. It reframes assessment not simply as a diagnostic task, but as a space for self-exploration and growth. Empirical studies have supported TA’s effectiveness across a range of populations, including adults (Durosini et al., 2017), couples (Provenzi et al., 2017), families (Tharinger et al., 2022), and children (Fantini et al., 2013). A recent meta-analysis affirmed TA’s impact on reducing distress, enhancing self-esteem, and increasing insight, highlighting its capacity to motivate individuals to engage with psychological or psychiatric treatment (Durosini and Aschieri, 2021). This growing interest in the therapeutic potential of assessment coincides with an important development in the broader field of intervention: the increasing implementation of Single-Session Interventions (SSIs), including Single-Session Cognitive Behavioral Therapy (SS-CBT) (Schleider, 2020; Talmon, 1990). SSIs can be defined as structured programs that consist of only one visit and apply cognitive-behavioral strategies (i.e., Cognitive restructuring). While CBT is usually delivered in multisession formats, research shows that for some individuals, particularly those facing structural or motivational barriers, a single clinical encounter can improve symptomatology and promote access to care (Cohen et al., 2023). Additionally, as Schleider and Fox (2025) report in their review including four-hundred-fifteen clinical trials, SSIs have shown small-to-moderate but reliable effects across a wide range of outcomes, including anxiety, depression, substance use, and service engagement. These findings hold across youth and adult populations, with evidence supporting both provider-led and self-guided delivery.

Considering that both TA and SS-CBT are designed to enhance access to care and facilitate the timely initiation of intervention, their integration offers a compelling model for contemporary clinical practice. TA fosters early engagement through collaborative meaning-making and emotional resonance, while SS-CBT provides focused, evidence-based strategies that can be delivered efficiently within a single session. Together, they offer a structured response to systemic barriers such as limited resources, long waitlists, and early dropout, providing an approach that is both clinically meaningful and practically feasible, even within research trials. To date, TA and SS-CBT have not yet been formally combined in either clinical protocols or empirical studies.

Building upon the theoretical and empirical foundations of TA and SS-CBT, the present study explored their application as a standalone intervention for a specific and underrepresented clinical population: adult women who carry the *FMR1* premutation (PM), a genetically at-risk group with heightened vulnerability to mood, anxiety, and cognitive difficulties collectively referred to as Fragile X-associated Neuropsychiatric Disorders (FXAND). Despite this risk profile, tailored psychological assessments for premutation carriers (PCs) remain limited. Here, we introduce FRAX-TA (FRAgile X-Therapeutic Assessment), a TA-based protocol designed specifically for PCs, combining the interpretive depth of TA with the therapeutic immediacy of SS-CBT.

**FIGURE 1:**
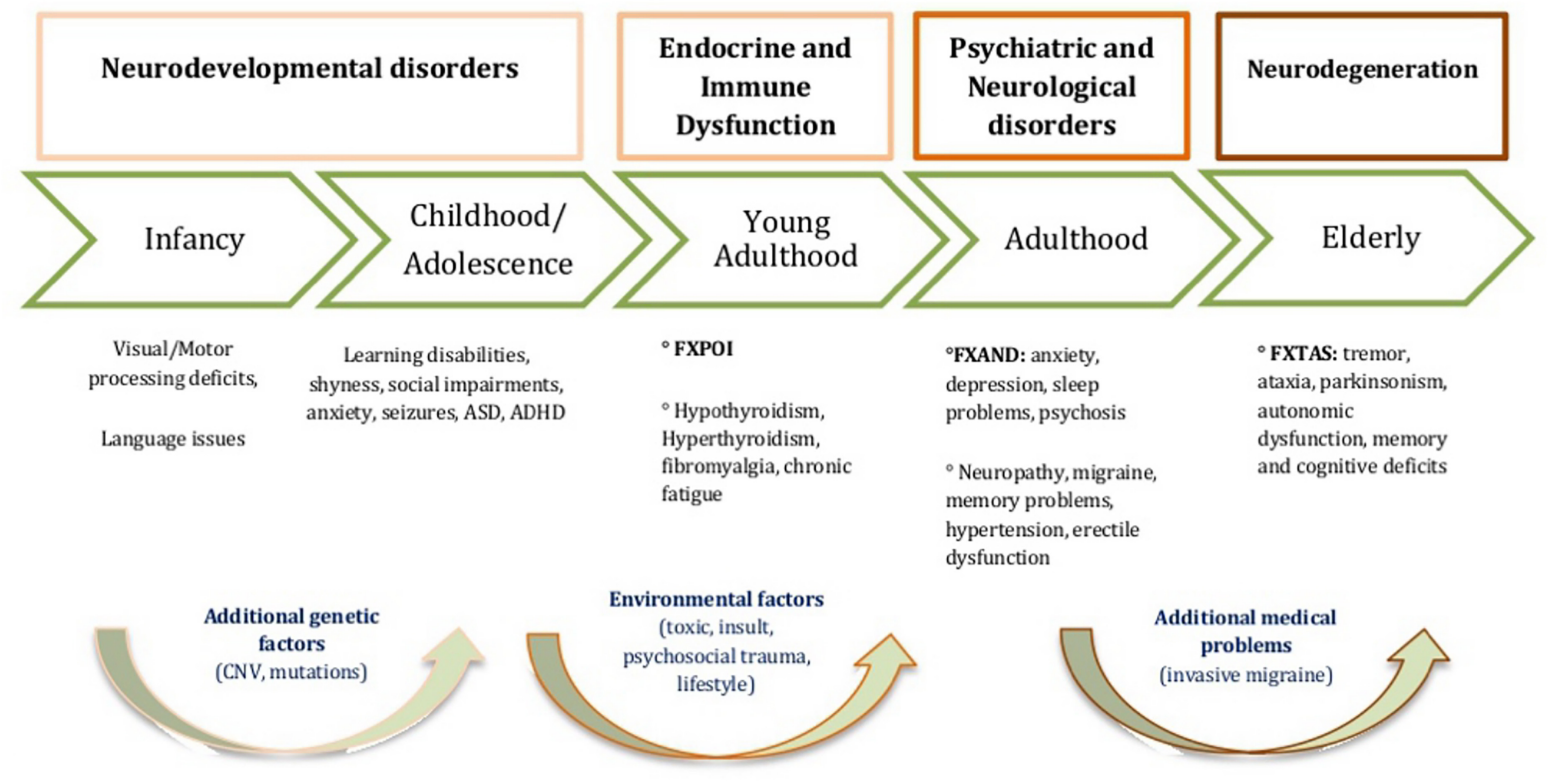
Clinical conditions associated to *FMRI* Premutation. Fragile X Premutation Associated Conditions (FXPAC) across lifespan. FXPOl, Fragile X-associated Primary Ovarian Insufficiency; FXAND, Fragile X- associated Neuropsychiatric Disorders; FXTAS, Fragile X-associated Tremor/Ataxia Syndrome. Adapted from Hagerman and Hagerman (2020).

The PM is a quite common condition affecting approximately 1 in 200 women and 1 in 400 men (Tassone et al., 2012, 2023). These individuals (mostly mothers) are often parents of children with Fragile X Syndrome (FXS), the most common inherited cause of intellectual disability and autism spectrum disorder (Richter and Zhao, 2021). People with PM typically present with 55–200 CGG repeats in the *FMR1* gene and elevated FMR1 mRNA levels in peripheral blood, leading to RNA toxicity (Tassanakijpanich et al., 2021). This mechanism underlies a group of conditions known collectively as Fragile X Premutation Associated Conditions (FXPAC), whose clinical presentation varies by age and developmental stage (Figure 1; adapted from Hagerman and Hagerman, 2020). Within FXPAC, the most widely recognized conditions are Fragile X-associated Tremor/Ataxia Syndrome (FXTAS) and Fragile X-associated Primary Ovarian Insufficiency (FXPOI), which affect approximately 16–20% of women with PM (Fink et al., 2018; Cabal-Herrera et al., 2020; Tassone et al., 2023). More recently, researchers have identified a separate cluster of symptoms, such as anxiety, depression, ADHD, fatigue, chronic pain, and pragmatic language difficulties that have been classified as FXAND (Hagerman et al., 2018; Cross et al., 2016). These conditions affect up to 50% of PCs and may begin in childhood, even in women without children with FXS (Roberts et al., 2009; Gossett et al., 2016; Aishworiya et al., 2022), supporting the view that FXAND is independent of caregiving status.

Despite growing recognition of FXPAC, the psychological profile of PCs remains understudied and clinically under-addressed. Many PCs report difficulties accessing informed care or finding professionals who understand the full range of PM-related manifestations (Protic and Hagerman, 2024; Espinel et al., 2016; Tassone et al., 2023). In Italy, these barriers are exacerbated by a lack of local expertise in adult PM presentations and limited availability of tailored support. To explore these issues, a prior pilot study investigated the treatment needs through an anonymous survey of Italian PCs, revealing significant psychological distress, particularly in the areas of anxiety and memory. The ninety-six respondents also emphasized the need for individualized treatments focused on anxiety and mood-related symptoms (Montanaro et al., 2024a). However, many participants demonstrated limited familiarity with relevant clinical terminology, such as FXAND, despite describing symptoms consistent with its criteria. This discrepancy likely reflects a combination of low health literacy and perceived stigma, both of which may delay diagnosis and limit access to appropriate care. These findings underscore the urgent need for clinical projects that pursue a dual aim: first, to collect more precise data on the psychological and neuropsychiatric characteristics of individuals with PM within the Italian context and second, to extend beyond diagnostic clarification by actively fostering psychological engagement and facilitating access to care. While the combination of TA and SS-CBT offer a compelling model for both purposes, their applicability to the PM population has not yet investigated.

## 2 Aim of the study

The main aim of the present study was to evaluate the impact of an adapted TA protocol on the psychological wellbeing of Italian female PCs. The resulting protocol, named FRAX-TA, included a session called the Psychoeducational Assessment (PA), in which standard TA techniques were integrated with strategies from the SS-CBT. Specifically, TA provided the collaborative interpretive process and diagnostic feedback, whereas SS-CBT introduced brief cognitive-behavioral tools aimed at promoting insight, cognitive flexibility, and adaptive coping. The SS-CBT strategies primarily drew on cognitive restructuring (Beck, 2016) as well as third-wave CBT principles such as psychological flexibility, values clarification, and acceptance-based coping (Hayes, 2016). The PA was delivered by a CBT therapist with expertise in the FXS and PM field (FAMM).

This design also allowed us to investigate whether the timing of the PA session, relative to baseline symptomatology, influenced specific psychological outcomes. To this end, participants were randomly assigned to four groups. While all participants completed the baseline questionnaires, cognitive assessments and biweekly follow-ups at the same stages, the timing of the PA session varied: it occurred approximately four (group A), six (group B), eight (group C), or ten (group D) weeks after baseline. This staggered design enabled us to differentiate between (1) the general therapeutic effects of FRAX-TA, which encompassed repeated engagement, structured assessments, and facilitated self-reflection on one’s symptoms and (2) the specific impact of the PA session itself.

Specifically, we tested three main hypotheses:

*H1: The long-term effect of FRAX-TA process:* We expected an overall improvement in the outcomes as a function of intervention engagement (i.e., comparing baseline and follow-up phases).

*H2: Middle-term effect of FRAX-TA process following PA:* We hypothesized that the most significant improvement would occur immediately after the PA session which is delivered in the middle of the FRAX-TA process (i.e., comparing pre-PA session and post-PA session).

*H3: PA delivery week effect:* Earlier exposure to the PA session (Groups A and B) would be associated with more sustained psychological gains than later sessions (Groups C and D).

To our knowledge, this is the first empirical study to investigate the therapeutic potential of integrating TA with SS-CBT in individuals with the PM. The findings carry implications for both research and clinical practice. First, a structured psychological assessment can serve as a meaningful intervention, particularly in populations with limited access to care, improving patients’ capacity to effectively communicate their symptoms and their general mental wellbeing. Second, understanding the optimal timing of the SS-CBT based on previous assessment sessions may enhance both its emotional impact and its diagnostic effectiveness, offering valuable guidance for best practices in complex clinical contexts.

## 3 Materials and methods

### 3.1 Participants

Participants were recruited through flyers posted by the Italian Fragile X Syndrome Association, as well as through informal peer referral among individuals already enrolled in the study. PCs who wished to participate could contact the Association via email, after which they were contacted by the research team for enrollment and provided written informed e-consent.

Inclusion criteria were as follows:

1. Being a carrier of the PM, as confirmed by genetic testing indicating the number of CGG repeats on the *FMR1* gene.
2. Being of legal age (18 years or older).
3. Being a resident of Italy.
4. Having a stable internet connection, as the study was conducted entirely online to allow participation of individuals from across the country.

The recruitment announcement was initially addressed to all birth genders; however, since only four males expressed interest in participating, the project was subsequently limited to women to ensure feasibility and methodological robustness. A total of 88 women responded to the flyer. Of these, nine underwent genetic testing again to determine the exact number of CGG repeats and were thus included. Conversely, three middle-aged volunteers were excluded because, although they believed they carried the PM, they were found to have the full mutation (FXS). Two participants withdrew from the study immediately after enrolment, and two others were excluded due to a lack of confirmed genetic testing. All the excluded participants were personally contacted by the CBT therapist, who provided a clear and respectful explanation of the reasons for their exclusion. The final sample therefore consisted of 81 women. Table 1 shows the main sociodemographic and clinical features at time of the recruitment. The information was collected through a Google Form distributed via e-mail to each participant. The form was designed to obtain electronic informed consent (e-consent) and gather key sociodemographic data. Participants were also asked to complete a set of self-report questionnaires regarding their psychological symptoms, any previous diagnoses, and their children’s behaviour. The average age of participants was 50.5 ± 9.41 years, with the majority falling within the 51–65 years age range (35.8%); 43.2% of participants had completed high school, and 85% had at least one child with FXS. The average number of CGG repeats was 90.5 ± 22.35. The 63% of participants denied any FXPAC diagnosis, while 24 out of 81 participants were diagnosed with FXPOI, and 5 with FXTAS. Only three participants recognized themselves as having any FXAND condition at the time of enrolment.

**TABLE 1:** Socio-demographic and clinical features at time of recruitment.

Variable	Level		Frequency (*N* = 81)	Percentage (%)	Proportion
Geographic area	Central Italy		39	48.1%	0.481
	Northern Italy		25	30.9%	0.309
	Southern Italy		17	21.0%	0.21
Education	Primary school or less		1	1.2%	0.012
	Middle school		6	7.4%	0.074
	High school		35	43.2%	0.432
	Bachelor’s degree		11	13.6%	0.136
	Master’s degree		19	23.5%	0.235
	Postgraduate Program		8	9.9%	0.099
	PhD or more		1	1.2%	0.012
Marital status	Married		56	69.1%	0.691
	Divorced		12	14.8%	0.148
	Single		9	11.1%	0.111
	In a relationship		4	4.9%	0.049
Child status	Children with FXS		69	85%	0.852
	Children without FXS		9	11%	0.037
	No children		3	4%	0.111
Income	≤ 15,000 euros		13	16.0%	0.16
	15,001–28,000 euros		31	38.3%	0.383
	28,001–50,000 euros		22	27.2%	0.272
	≥ 50,000 euros		5	6.2%	0.062
	Prefer not to answer		10	12.3%	0.123
FXPAC diagnosis (self-report)	NONE		51	63.0%	0.63
	FXPOI		22	27.2%	0.272
	FXTAS		3	3.7%	0.037
	FXAND		3	3.7%	0.037
	FXPOI + FXTAS		2	2.5%	0.025
Age range	26–40		17	21.0%	0.21
	41–50		27	33.3%	0.333
	51–65		29	35.8%	0.358
	66+		8	9.8%	0.09
**Descriptive**	**Mean**	**Med**	**SD**	**Min**	**Max**
Age CGG rep	50.5	48.7	9.41	31.7	81.0
	90.5	85	22.35	57	189

Med, median; SD, standard deviation; Min, minimum; Max, maximum; FXPOI, Fragile X-associated Primary Ovarian Insufficiency; CGG (cytosine, guanine, guanine) repeats. FXAND, Fragile X-associated Neuropsychiatric Disorders; FXTAS, Fragile X-associated Tremor/Ataxia Syndrome. FXPAC diagnosis, Fragile X Premutation Associated Conditions diagnosed prior of the study.

### 3.2 Procedure

Recruitment began in June 2024 and concluded in July 2024. Eighty-eight women who self-identified as having the PM expressed interest and were added to a contact list that remained open for 1 month. As part of initial registration, participants provided consent and contact details. All individuals were contacted via phone by the CBT therapist performing the PA, who informed them that they would soon be reached by one of two trainees (hereafter referred to as Assessor A and B). Assessors subsequently reached out to collect demographic and clinical data through a Google Form, used to confirm eligibility and assign participants to one of four experimental groups. Randomization occurred immediately after registration closure, given that most participants had been referred through the Italian Fragile X Syndrome Association, which assured a high likelihood of confirmed PM status. This approach allowed any rare misunderstandings about genetic status to be addressed compassionately during subsequent contact, rather than through pre-screening requirements. Each group differed only in the timing of their PA with the CBT therapist:

- Group A (*n* = 22): benefitted of the PA after 4 weeks from T0
- Group B (*n* = 21): benefitted of the PA after 6 weeks from T0
- Group C (*n* = 18): benefitted of the PA after 8 weeks from T0
- Group D (*n* = 20): benefitted of the PA after 10 weeks from T0

It is important to note that the groups were initially balanced through random assignment; however, differences in final group sizes reflect attrition following enrollment. Within each group, participants were evenly distributed between Assessor A and Assessor B. Notably, both assessors received comprehensive training prior to the start of the study to ensure consistent application of standardized assessment procedures. Furthermore, preliminary checks were conducted to assess potential experimenter effects; no effect was found.

To ensure continuity and minimize variability in administration, each participant was consistently followed by the same assessor throughout the entire duration of the study. This approach was adopted not only to enhance methodological rigor and ensure consistency in data collection procedures, but also to foster a stable therapeutic connection, thereby promoting participant engagement and trust over time.

The *FRAX-TA protocol* served as the core framework for data collection and participant engagement across all groups (see “Structure of the Fragile X Therapeutic Assessment” below and Supplementary Appendix 1).

As shown in Table 2, the timeline included:

**TABLE 2:** Timeline of FRAX-TA process.

Group	T0 (week 0)	T1 (week 2)	T2 (week 4)	T3 (week 6)	T4 (week 8)	T5 (week10)	T6 (week 12)	T7 (week 14)
**A** (mean ± SD age: 50.5 ± 11.7)	Google Form via e-mail + phone CBA-VE_pre	Zoom CBA-VE_pre + Zoom cognitive screening	Zoom PA with CBT therapist + CBA-VE_afterPA	Phone CBA-VE_post	Phone CBA-VE_post	Phone CBA-VE_post	Phone CBA-VE_post	Phone CBA-VE_post
**B** (mean ± SD age: 51.2 ± 10.6)	Google Form via e-mail + phone CBA-VE_pre	Zoom CBA-VE_pre + Zoom cognitive screening	Phone CBA-VE_pre	Zoom PA with CBT therapist + CBA-VE_afterPA	Phone CBA-VE_post	Phone CBA-VE_post	Phone CBA-VE_post	Phone CBA-VE_post
**C** (mean ± SD age: 51.2 ± 9)	Google Form via e-mail + phone CBA-VE_pre	Zoom CBA-VE_pre + Zoom cognitive screening	Phone CBA-VE_pre	Phone CBA-VE_pre	Zoom PA with CBT therapist + CBA-VE_afterPA	Phone CBA-VE_post	Phone CBA-VE_post	Phone CBA-VE_post
**D** (mean ± SD age: 49.2 ± 4.9)	Google Form via e-mail + phone CBA-VE_pre	Zoom CBA-VE_pre + Zoom cognitive screening	Phone CBA-VE_pre	Phone CBA-VE_pre	Phone CBA-VE_pre	Zoom PA with CBT therapist + CBA-VE_afterPA	Phone CBA-VE_post	Phone CBA-VE_post

CBA-VE_pre, All CBA-VE assessments administered prior to the PA session. CBA-VE_afterPA, CBA-VE assessments administered immediately after the PA session. CBA-VE_post, All CBA-VE assessments administered during the sessions following the PA. SD, standard deviation. Groups were comparable in socio-demographic features (*p* > 0.05, Supplementary Tables 1, 2).

- T0: All participants, across all groups, completed the same baseline procedures. These consisted of an online consent, demographics, a set of questionnaires (administered as part of a broader project aimed at characterizing the cognitive-behavioral profile of Italian women with the PM, and detailed in Montanaro et al., 2026), and the first phone-administered Cognitive Behavioral Assessment for Outcome Evaluation (Cognitive Behavioral Assessment—Valutazione dell’esito, CBA-VE; available on-line on https://www.ordinepsicologier.it/public/genpags/bigs/protocollo_CBAVE.pdf, Michielin et al., 2008).
- T1: Also identical across all groups, this session consisted of a Zoom-based cognitive screening preceded by a second administration of the CBA-VE in the same session. This order was chosen to minimize potential interference from performance anxiety on CBA-VE scores. This session was performed after 2 weeks from T0.
- T2-T7: Ongoing CBA-VE assessments every 2 weeks, with the PA conducted according to group assignment, specifically, at T2 for Group A (after 4 weeks), T3 for Group B (after 6 weeks), T4 for Group C (after 8 weeks), and T5 for Group D (after 10 weeks). To note, during each PA session, the CBA-VE was administered at the end of the interview to assess any immediate effects of PA on emotional and psychological functioning.

Although the CBA-VE is a self-report questionnaire, we chose to administer it via phone or zoom to maintain participant engagement and foster a sense of connection. More importantly, the CBA-VE enabled us to monitor participants’ psychological wellbeing every 2 weeks throughout the study. At the end of the protocol, participants were invited to complete an anonymous feedback questionnaire. This final step was intended to gather participants’ impressions of the overall experience and inform future research design and clinical practices. The entire study concluded in March 2025.

### 3.3 Structure of the Fragile X Therapeutic Assessment

To support the implementation of the FRAX-TA protocol, the CBT therapist provided targeted training to two trainees (Assessors A and B). Training included background information on FXS, the PM, related psychological and medical challenges, and detailed procedural instructions. Assessors also attended selected sessions of a combined neuropsychological and cognitive-behavioral group therapy for adults with FXS (Montanaro et al., 2023, 2024b), which provided additional exposure to the clinical phenotype and the lived experience of individuals with Fragile X-related conditions.

Once readiness was confirmed, the FRAX-TA protocol was structured into six sequential phases. A detailed description of the procedures implemented during each phase of the protocol is provided in Supplementary Appendix 1.

1. Welcoming Phase (WP)

The first contact with participants was conducted directly by the CBT therapist by phone, followed by a written summary sent via e-mail. This approach aimed to establish an initial therapeutic connection, clarify the study procedures, and provide participants with a direct communication channel with the therapist. Each WP session lasted approximately 15 min.

2. Assessment Onboarding Phase (AOP)

Following the WP, participants were contacted by one of the trained assessors. During this phase, informed consent and demographic information were collected through an online form, and the first administration of the CBA-VE was conducted via semi-structured telephone interview. Participants were also informed about the scheduling of subsequent sessions. Each AOP lasted approximately 20 min.

3. Cognitive Screening Phase (CSP)

The CSP was conducted via Zoom and included a brief psychological check-in followed by cognitive screening. Participants completed the Raven Standard Progressive Matrices (Raven, 2008) and the Telephone Montreal Cognitive Assessment (T-MoCA) (Katz et al., 2021; Wang et al., 2023). The aim of this phase was to obtain baseline cognitive information that could serve as a reference point for future clinical monitoring. Each CSP session lasted approximately 60 min.

4. Psychoeducational Assessment (PA)

The PA represented the core therapeutic component of the FRAX-TA protocol and was conducted by the CBT therapist. Sessions lasted approximately 100–120 min and integrated elements of TA, psychoeducation, and a brief CBT-informed intervention. The session included a semi-structured clinical interview exploring participants’ personal experiences with the PM, psychodiagnostic evaluation using the Structured Clinical Interview for DSM-5 Disorders (SCID-5), feedback on cognitive screening results, and collaborative interpretation of the assessment findings. Psychoeducational and CBT-informed strategies (i.e., cognitive restructuring) were used to promote symptom awareness, psychological flexibility, and adaptive coping. In addition, the therapist introduced evidence-based integrative health strategies for individuals carrying the PM, including recommendations related to nutrition, exercise, and lifestyle modifications. Additionally, at the end of the session, a detailed summary e-book prepared by the therapist was sent to participants via e-mail (Supplementary Appendix 2). Finally, the CBA-VE was administered at the end of the session to evaluate immediate psychological responses.

5. Follow-up Monitoring (Phone CBA-VE)

After the PA session, participants continued completing the CBA-VE every 2 weeks until the end of the protocol. Assessments were administered by telephone by the trained assessors and lasted approximately 15–20 min.

6. Anonymous Feedback Questionnaire

At the conclusion of the study, participants were invited to complete an anonymous online questionnaire designed to gather feedback on the research experience, perceived emotional support during the protocol, and suggestions for future research and clinical interventions.

### 3.4 Measures

#### 3.4.1 Quantitative measures

The Cognitive Behavioral Assessment for Outcome Evaluation (CBA-VE) was used to monitor the effectiveness of the FRAX-TA protocol (Michielin et al., 2008). This self-report instrument includes 80 items, each rated on a 5-point Likert scale (1 = “nothing” to 5 = “a lot”), designed to assess participants’ psychological experiences over the preceding 2 weeks. Although the CBA-VE is typically self-administered, to enhance participant engagement and support the therapeutic alliance, it was administered by Assessor A, Assessor B, or the CBT therapist during scheduled Zoom or telephone sessions (see Table 2).

The CBA-VE consists of five distinct subscales:

- *Anxiety* (14 items; e.g., “I have been upset about trivial things”),
- *Well-Being* (15 items; e.g., “I have done things that interested and involved me”),
- *Perception of Positive Change* (11 items; e.g., “I have tried to deal with difficulties rather than avoid them”),
- *Depression* (19 items; e.g., “I have been tormented by feelings of guilt”), and
- *Psychological Distress* (21 items; e.g., “I have felt debased or mocked”).

In line with the CBA-VE’s recommended temporal frame and to track psychological trends over time, participants completed the measure approximately every 2 weeks throughout the study, resulting in eight repeated assessments. The CBA-VE has demonstrated strong psychometric properties, including high internal consistency (Cronbach’s α = 0.74–0.91 in normative samples), good reliability, and robust structural validity across its five interrelated domains. It has also shown good capacity to discriminate between clinical and non-clinical populations. Scores can be interpreted using normative data available for both clinical and non-clinical samples. Given that individuals with the PM do not always exhibit psychological symptoms, we opted to use non-clinical normative data for score correction.

For enhanced interpretability, we computed T-scores for each subscale using the formula:

*T = 50 + 10 × [(raw score - subscale mean)/subscale standard deviation]* (Michielin et al., 2008; Sanavio et al., 2013; Bertolotti et al., 2015).

#### 3.4.2 Qualitative measures

At the end of the study, participants were invited to complete an anonymous semi-structured questionnaire via Google Form, specifically developed for this research project. The instrument was designed to gather information on overall satisfaction with the research experience, clarity of instructions, perceived support from the research team, personal relevance of the sessions, and suggestions for future investigations (Supplementary Appendix 3). While the questionnaire has not undergone formal validation, it was intended to provide preliminary, exploratory insights into participant experiences and inform the refinement of future protocols.

## 4 Research design and statistical analysis

FRAX-TA consisted of an eight-session longitudinal therapeutic protocol, including one “single-session therapy” module, named PA. This module was delivered at different time points across four distinct groups: specifically, in the 4th (group A), 6th (group B), 8th (group C), or 10th (group D) week after T0. See Table 2 for the Study Timeline. A mixed-design Bayesian Repeated Measures ANOVA was employed, with a 4 (PA delivery week, referring to the week when the PA was delivered during the overall intervention period: 4th, 6th, 8th or 10th week, between-subjects) × 3 (Phase of FRAX-TA: CBA-VE_pre; CBA-VE_afterPA; CBA-VE_post, within-subjects) factorial structure.

It is important to note that the lengths of the CBA-VE_pre and CBA-VE_post phases varied across the four groups due to the different timing of the single-session therapy. Specifically:

- The 4th week group included two baseline sessions, one intervention week and 5 follow-up sessions.
- The 6th week group had 5 baseline sessions, one intervention week and two follow-up sessions.
- The remaining groups followed intermediate patterns.

To account for this, the baseline and follow-up values were computed as means of the respective sessions, while the post-intervention session corresponded to the single time in which the PA was delivered. This aggregation approach allows comparisons across groups despite differences in phase length. This methodological structure is conceptually inspired by multiple-baseline single-case designs, which deliberately vary the number of baseline observations before the intervention to examine treatment effects across staggered starting points (Kazdin, 2011).

We adopted a Bayesian approach to the ANOVA (Rouder et al., 2012) to directly estimate the likelihood of each model obtained by the independent variables included in the analysis and aimed to verify the three key hypotheses listed in the paragraph “Aim of the Study.”

The selected model was, in general, the best-fitting model as determined by Bayesian model comparison criteria. The Jeffreys’ scale for the interpretation of Bayes factors (BFs) for each model was used (e.g., Lee and Wagenmakers, 2013). To decide to interpret also models worse than the best one, we calculate the ratio between the best and the subsequent model BFs. Since there isn’t a direct index for ratios between model BFs, Jeffreys’ scale can provide context. A BF ratio, for example, between the best model and another model, can be interpreted in terms of *how much more probable the best model is compared to the other*. If this ratio is very high (e.g., > 100), it means the worse model has *negligible evidential support* compared to the best one. On the other hand, a BF Ratio under 3 it suggests there is *little difference in evidence* between the two models. In this case, it might be reasonable to interpret both models, or at least consider that the worse model offers an almost equally plausible alternative. This can be particularly relevant if the worse model is more parsimonious or theoretically more significant, in some way. Following the examples, any model compared to the best showing a ratio (best model/other model) larger than 10 can be neglected.

## 5 Results

### 5.1 Baseline comparisons—Bayesian one-way ANOVA

Descriptives are included in Supplementary Tables 3–7. To assess group differences at baseline in all the dimensions of the CBA-VE, we conducted five Bayesian ANOVAs with Week of the PA as the between-subject factor. The resulting Bayes Factors (BF_10_) were used to quantify the evidence in favor of the alternative hypothesis relative to the null. For *anxiety* and *depression*, the analyses provided substantial evidence in favor of the null model, indicating no relevant baseline differences among the four groups. Similarly, no differences were observed for *perceived change* and *wellbeing*, with the null model clearly preferred in both cases. For *psychological distress*, findings showed only anecdotal evidence over the null (BF_10_ = 1.36). *Post hoc* comparisons suggested moderate evidence for higher distress levels in participants from the 4th week compared to those from the 6th (BF_10_ = 2.52) and 8th (BF_10_ = 2.33) weeks. However, descriptive statistics (Supplementary Table 3) showed that group means were closely aligned, and these differences remained subclinical in magnitude (group A: *M* = 55.3; SD = 10.81; group B: *M* = 48.3; SD = 8.18; group C: *M* = 48.4; SD = 7.43; group D: *M* = 59.9; SD = 12.61).

### 5.2 Bayesian repeated measures ANOVA

#### 5.2.1 Anxiety

Table 3 lists the five different models under consideration. The BF^10^ column shows that, compared to the Null model, two models receive overwhelming support from the data. The model that receives the most support against the Null model is main effect model with *Phase of FRAX-TA* (BF_10_ = *411.416*). The model with the two main effects *Phase of FRAX-TA* + *Group (A-D, based on the PA delivery week)* decreases the degree of this support by a factor of 3.51. The model adding the interaction decreases the degree of this support with respect to the best model by a factor of 18.47. In synthesis, the second model showed a moderate difference with the best and it required to be reported. The third model, conversely, can be neglected. Finally, for further assistance in model selection, we performed a Bayesian Model Averaging (BMA; Etz and Wagenmakers, 2017), which aims to circumvent model selection uncertainty. From this additional analysis, it is evident that the specific contribution of the second variable (*Group*) is negligible. Therefore, for the interpretation of this analysis, we will only consider the model with the variable (*Phase of FRAX-TA*) (Table 4).

**TABLE 3:** Comparisons of models with the null.

Models	P(M)	P(M| data)	BF_*M*_	BF_10_	Error %
Null model (incl. subject)	0.200	0.00181	0.00726	1.000	
Phase of FRAX-TA	0.200	0.74518	11.69724	411.416	0.980
Group	0.200	4.77e-4	0.00191	0.263	0.400
Phase of FRAX-TA + Group	0.200	0.21219	1.07733	117.148	0.847
Phase of FRAX-TA + Group + Phase of FRAX-TA*Group	0.200	0.04035	0.16818	22.277	1.089

All models include subject. P(M) is the uniform distribution of probabilities for each model, P(M | data) is the posterior probability of each model, BF_*M*_ is the change from prior to posterior odds of the model, BF_10_ is the Bayes factor comparing the model with the null. Finally, the error % is an index of variability of the estimation.

**TABLE 4:** Analysis of effects for anxiety.

Effects	P(incl)	P(incl| data)	BF_*Inclusion*_
Phase of FRAX-TA	0.600	0.9977	290.739
Group	0.600	0.2530	0.226
Phase of FRAX-TA*Group	0.200	0.0403	0.168

P(incl) is the prior inclusion probability for the effect, P(incl | data) is the posterior probability for the inclusion and, finally, the BFinclusion is the change from prior to posterior inclusion odds.

For *Phase of FRAX-TA*, the following post-hoc comparisons were observed (Individual comparisons are based on the default *t*-test with a Cauchy [0, r = 1/sqrt (2)] prior. The “U” in the Bayes factor subscript denotes that it is uncorrected):

- pre vs. post: BF_10, U_ = 70532.917, indicating extremely strong evidence for an improvement (reflecting a reduction of the score for the dimension of “anxiety”) between the phases pre- and post-.
- pre vs. post-PA: BF_10, U_ = 0.22, suggesting evidence against a difference between these phases.

These results support only H1 (overall improvement comparing the initial and final phases of FRAX-Ta) but did not support neither H2 (an improvement immediately following the PA session was not present) nor H3 (a difference in efficacy due to the earliness in delivering PA, that is, the SS-CBT session).

#### 5.2.2 Depression

Table 5 lists the five different models under consideration. The BF^10^ column shows that, compared to the Null model, two models receive overwhelming support from the data. The model that receives the most support against the Null model is the main effect model with Phase of FRAX-TA (BF_10_ = 14408.003). The model with the two main effects Phase of FRAX-TA + Group (A–D, based on the PA delivery week) decreases the degree of this support by a factor of 3.03. The model adding the interaction further decreases the degree of this support with respect to the best model by a factor of 15.57. In synthesis, the second model showed a moderate difference with the best and it required to be reported. The third model, conversely, can be neglected. Again, for further assistance in model selection, we performed a Bayesian Model Averaging (BMA; Etz and Wagenmakers, 2017). We found that the specific contribution of the second variable (Group) is negligible. Therefore, for the interpretation of this analysis, we will only consider the model with the variable (Phase of FRAX-TA) (Table 6).

**TABLE 5:** Comparisons of models with the null.

Models	P(M)	P(M| data)	BFM	BF_10_	Error %
Null model (incl. subject)	0.200	4.98e-5	1.99e-4	1.000	
Phase of FRAX-TA	0.200	0.7172	10.144	14408.003	1.787
Group	0.200	1.50e-5	5.98e-5	0.300	0.340
Phase of FRAX-TA + Group	0.200	0.2367	1.240	4754.344	0.969
Phase of FRAX-TA + Group + Phase of FRAX-TA*Group	0.200	0.0461	0.193	925.694	1.033

Nota. All models include subject. P(M) is the uniform distribution of probabilities for each model, P (M | data) is the posterior probability of each model, BF_*M*_ is the change from prior to posterior odds of the model, BF_10_ is the Bayes factor comparing the model with the null. Finally, the error % is an index of variability of the estimation.

**TABLE 6:** Analysis of effects for depression.

Effects	P(incl)	P(incl| data)	BFInclusion
Phase of FRAX-TA	0.600	0.9999	10298.715
Group	0.600	0.2828	0.263
Phase of FRAX-TA*Group	0.200	0.0461	0.193

P(incl) is the prior inclusion probability for the effect, P(incl | data) is the posterior probability for the inclusion and, finally, the BF inclusion is the change from prior to posterior inclusion odds.

For Phase of FRAX-TA, the following *post-hoc* comparisons were observed (Individual comparisons are based on the default *t*-test with a Cauchy [0, *r* = 1/sqrt (2)] prior. The “U” in the Bayes factor subscript denotes that it is uncorrected):

- pre vs. afterPA: BF_10_, _*U*_ = 1371.325, indicating extremely strong evidence for an early improvement (i.e., a reduction of the score for the dimension of “depression”) immediately after the PA session;
- pre vs. post: BF_10_, _*U*_ = 54274.824, indicating extremely strong evidence for an overall improvement;
- afterPA vs. post: BF_10_, _*U*_ = 0.128, suggesting evidence against a difference between these phases.

These results support H1 (overall improvement comparing the initial and final phases of FRAX-TA) and H2 (an improvement immediately following the PA session) but did not support H3 (a difference in efficacy due to the earliness in delivering PA, that is, the SS-CBT session).

#### 5.2.3 Psychological distress

Table 7 lists the five different models under consideration. The BF^10^ column shows that, compared to the Null model, three models receive extremely strong and comparable support from the data. Specifically, the models with (1) the main effect Phase of FRAX-TA (BF_10_ = 3.26 × 10^17^), (2) the two main effects Phase of FRAX-TA + Group (BF_10_ = 2.41 × 10^17^), and (3) the full model with the interaction (BF_10_ = 2.63 × 10^17^) are all favored over the Null model by a similar magnitude (differences between them are within a factor of ∼1.35). In such cases, model selection is informed by the analysis of effects. The inclusion analysis clearly indicates that only the effect of Phase of FRAX-TA contributes substantially to explaining the data, while Group and the interaction Phase of FRAX-TA * Group are marginal. Therefore, for the interpretation of this analysis, we will only consider the model with the variable (Phase of FRAX-TA) (Table 8).

**TABLE 7:** Comparisons of models with the null.

Models	P(M)	P(M| data)	BF_*M*_	BF_10_	error %
Null model (incl. subject)	0.200	1.21e-18	4.82e-18	1.000	
Phase of FRAX-TA	0.200	0.393	2.59	3.26e+17	0.907
Group	0.200	7.05e-19	2.82e-18	0.585	1.096
Phase of FRAX-TA + Group	0.200	0.291	1.64	2.41e+17	1.246
Phase of FRAX-TA + Group + Phase of FRAX-TA*Group	0.200	0.317	1.85	2.63e+17	1.995

Nota. All models include subject. P(M) is the uniform distribution of probabilities for each model, P(M | data) is the posterior probability of each model, BF_*M*_ is the change from prior to posterior odds of the model, BF_10_ is the Bayes factor comparing the model with the null. Finally, the error % is an index of variability of the estimation.

**TABLE 8:** Analysis of effects for psychological distress.

Effects	P(incl)	P(incl| data)	BF_*Inclusion*_
Phase of FRAX-TA	0.600	1.000	∞
Group	0.600	0.607	1.03
Phase of FRAX-TA*Group	0.200	0.317	1.85

P(incl) is the prior inclusion probability for the effect, P(incl | data) is the posterior probability for the inclusion and, finally, the BFinclusion is the change from prior to posterior inclusion odds.

For Phase of FRAX-TA, the following post-hoc comparisons were observed (Individual comparisons are based on the default t-test with a Cauchy [0, r = 1/sqrt (2)] prior. The “U” in the Bayes factor subscript denotes that it is uncorrected):

- pre vs. afterPA: BF_10_, _*U*_ = 1.44 × 10^13^, indicating extremely strong evidence for an early improvement (i.e., a reduction in “psychological distress”) immediately following the PA session;
- pre vs. post: BF_10_, _*U*_ = 1.94 × 10^10^, indicating extremely strong evidence for an overall improvement;
- afterPA vs. post: BF_10_, _*U*_ = 0.800, suggesting evidence against a difference between these phases.

These results support H1 (overall improvement comparing the initial and final phases of FRAX-TA) and H2 (an improvement immediately following the PA session) but did not support H3 (a difference in efficacy due to the earliness in delivering PA, that is, the SS-CBT session).

#### 5.2.4 Positive perception of change

Table 9 lists the five different models under consideration. The BF^10^ column shows that only one model—the model including both main effects and their interaction—receives moderate support from the data when compared to the Null model (BF_10_ = 4.358). All other models, including the one with only the Phase of FRAX-TA, show BF_10_ values close to 1, indicating no substantial evidence in favor of any simpler model. The effect analysis confirms the choice of the most complex model, as the interaction between Phase of FRAX-TA and Group contributes most strongly (BF_*Inclusion*_ = 6.27), whereas the main effects of Phase of FRAX-TA (BF_*Inclusion*_ = 2.79) and Group (BF_*Inclusion*_ = 1.69) are only marginally supported. Therefore, for the interpretation of this analysis, we will consider the model including Phase of FRAX-TA, Group, and their interaction (Table 10).

**TABLE 9:** Comparisons of models with the null.

Models	P(M)	P(M| data)	BF_*M*_	BF_10_	Error %
Null model (incl. subject)	0.200	0.1401	0.651	1.000	
Phase of FRAX-TA	0.200	0.1431	0.668	1.022	1.090
Group	0.200	0.0528	0.223	0.377	0.574
Phase of FRAX-TA + Group	0.200	0.0536	0.227	0.383	0.781
Phase of FRAX-TA + Group + Phase of FRAX-TA*Group	0.200	0.6104	6.266	4.358	1.281

Nota. All models include subject. P(M) is the uniform distribution of probabilities for each model, P(M | data) is the posterior probability of each model, BF_*M*_ is the change from prior to posterior odds of the model, BF_10_ is the Bayes factor comparing the model with the null. Finally, the error % is an index of variability of the estimation.

**TABLE 10:** Analysis of effects for psychological distress.

Effects	P(incl)	P(incl| data)	BF_*Inclusion*_
Phase of FRAX-TA	0.600	0.807	2.79
Group	0.600	0.717	1.69
Phase of FRAX-TA*Group	0.200	0.610	6.27

P(incl) is the prior inclusion probability for the effect, P(incl | data) is the posterior probability for the inclusion and, finally, the BFinclusion is the change from prior to posterior inclusion odds.

For Phase of FRAX-TA, the following post-hoc comparisons were observed (Individual comparisons are based on the default *t*-test with a Cauchy [0, r = 1/sqrt (2)] prior. The “U” in the Bayes factor subscript denotes that it is uncorrected):

- pre vs. post: BF_10_, _*U*_ = 11.463, indicating strong evidence for an overall improvement in the perception of change;
- pre vs. afterPA: BF_10_, _*U*_ = 0.128, suggesting evidence against a difference between these phases;
- afterPA vs. post: BF_10_, _*U*_ = 1.135, providing anecdotal evidence in favor of a difference.

Group comparisons yielded the following key results:

- Group B showed a higher final score compared to Group C (BF_10_, _*U*_ = 5.076), suggesting moderate evidence for a greater improvement in Group B;
- All other pairwise comparisons returned Bayes factors below 3, indicating anecdotal or weak evidence at best.

**FIGURE 2:**
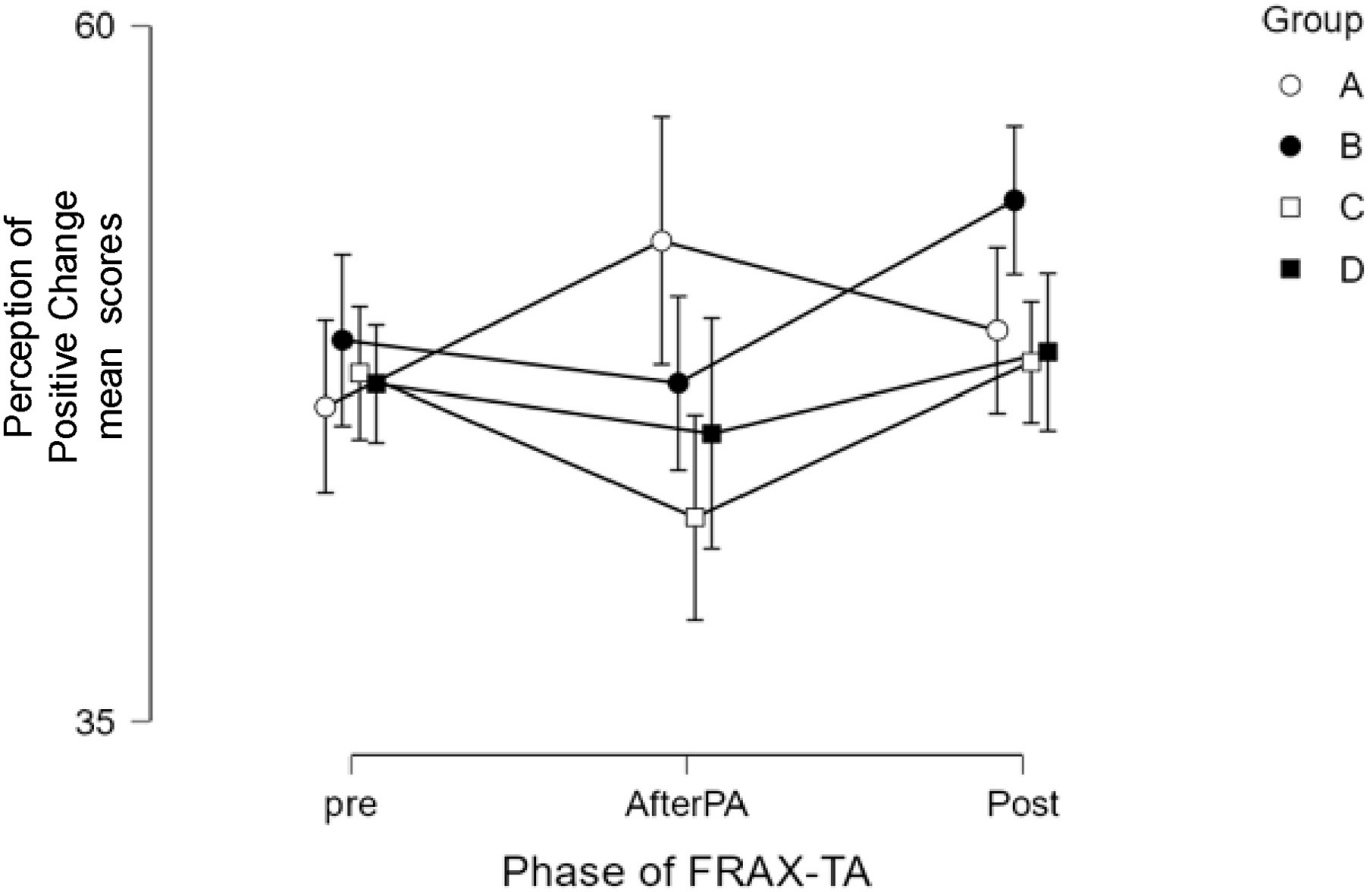
Descriptives Plot. Mean scores for Perception of Positive Change across the three phases of the FRAX-TA protocol (pre, AfterPA, post) for Groups A–D. Error bars represent standard errors.

These results support H1 (overall improvement across phases of FRAX-TA) and H2 (differential trends between groups, with some indication of early change), but not H3 (systematic differences based on timing of SS-CBT delivery). The interaction effect reflects a non-uniform pattern of change across groups (Figure 2).

#### 5.2.5 Wellbeing

Table 11 lists the five different models under consideration. The BF^10^ column shows that none of the models receive substantial support compared to the Null model. The model with the main effect of Phase of FRAX-TA has a BF_10_ = 0.5806, while all other models display even lower Bayes factors, indicating no evidence in favor of any effect related to Phase, Group, or their interaction. The Null model remains the most supported by the data (BF_10_ = 1).

**TABLE 11:** Comparisons of models with the null.

Models	P(M)	P(M| data)	BF_*M*_	BF_10_	Error %
Null model (incl. subject)	0.200	0.4350	3.0800	1.0000	
Phase of FRAX-TA	0.200	0.2526	1.3518	0.5806	2.537
Group	0.200	0.1901	0.9389	0.4370	0.757
Phase of FRAX-TA + Group	0.200	0.1107	0.4980	0.2545	1.747
Phase of FRAX-TA + Group + Phase of FRAX-TA*Group	0.200	0.0116	0.0468	0.0266	0.975

Nota. All models include subject. P(M) is the uniform distribution of probabilities for each model, P(M | data) is the posterior probability of each model, BF_*M*_ is the change from prior to posterior odds of the model, BF_10_ is the Bayes factor comparing the model with the null. Finally, the error % is an index of variability of the estimation.

These results suggest that the intervention did not modify participants’ perception of general wellbeing. Hypothesis H1 (a global change in well-being across the FRAX-TA phases) is not supported.

### 5.3 Anonymous feedback questionnaire

Sixty-two participants responded to the online anonymous feedback questionnaire. Overall, feedback was highly positive across all dimensions assessed (percentages and main results are presented in Supplementary Appendix 1). Regarding the close-ended questions (1–7), most respondents (58.1%) reported being extremely satisfied with their overall experience in the study, with an additional 33.9% indicating they were very satisfied. In terms of feeling supported and respected, 66.1% of participants selected “extremely,” and 27.4% selected “very,” suggesting a strong perception of psychological safety and respect throughout the process. Regarding the impact of participation, 40.3% felt the experience helped them significantly in some aspect of their lives, while 33.9% reported a moderate benefit. The PA session received particularly high ratings: 67.7% of participants reported being extremely satisfied, and 24.2% reported being very satisfied. Similarly, the sessions conducted by the two assessors were rated extremely positively by 58.1% of participants, with 30.6% selecting “very satisfied.” There was also strong enthusiasm for the continuation of the research project, with 67.7% expressing an extreme desire for it to proceed in the coming years, and an additional 27.4% indicating they would “very much” like to see it continue.

Main responses to the open-ended questions (8–11) are presented in Supplementary material. Overall, participants reported that the experience fostered greater self-awareness, helped them better understand their emotional and relational patterns, and provided concrete strategies for managing anxiety and supporting family members with FXS. Many highlighted the value of being listened to in a nonjudgmental and empathic environment, and several described the research as a unique opportunity to feel seen, supported, and informed about a condition that is often underrecognized. Participants also offered constructive suggestions for future research, including expanding psychological support, involving family members, promoting peer dialogue, and addressing broader health concerns related to carrier status.

## 6 Discussion

The present study sought to evaluate the potential therapeutic effects of a tailored TA protocol combined with a SS-CBT intervention, referred to as FRAX-TA, specifically developed for PCs. Using a mixed-methods design we investigated whether FRAX-TA could produce measurable improvements in emotional functioning. Additionally, we examined how the timing of the PA session with the CBT therapist influenced these outcomes, and whether participants perceived the overall assessment process as meaningful and supportive. To our knowledge, this is the first empirical study to systematically explore the feasibility and effectiveness of a combined TA and SS-CBT model tailored to this genetically at-risk population. The main findings can be summarized according to initial hypotheses:

*H1*: Long-term effect of FRAX-TA process

One of the study’s main objectives was to assess whether participants would demonstrate progressive psychological improvement over time. Results suggest that FRAX-TA produced both immediate and sustained therapeutic effects. Reductions in depression, psychological distress, and anxiety scores on the CBA-VE were observed. Across all three of these domains, participants demonstrated improvements from baseline (CBA-VE_pre) to follow-up (CBA-VE_post), indicating that the FRAX-TA protocol was effective in reducing emotional symptoms. The magnitude of Bayes Factors, especially in depression and psychological distress, provides support for the presence of an intervention effect. This supports the evidence that the psychological benefits were not only tied to the timing of the PA session, but rather to the overall FRAX-TA process, characterized by regular check-ins and relational continuity. Notably, the most consistent improvements emerged in psychological distress, underscoring the cumulative impact of ongoing supportive contact. These findings are consistent with prior literature indicating that assessment, when delivered collaboratively and with therapeutic intent, can act as an intervention in itself (Finn and Tonsager, 1992; Durosini and Aschieri, 2021). In this study, the emotional support embedded in the assessment process, as reflected in participants’ feedback, likely enhanced perceived validation and engagement. This is especially relevant given that the therapeutic value of clinical research is often underestimated by researchers (Dimidjian and Hollon, 2010) and underappreciated by participants in non-compensated studies (Kazdin, 2021).

Considering the Perception of Positive Change subscale, the evidence was supportive but moderate. Improvements were observed primarily between CBA-VE_pre and CBA-VE_post, rather than immediately after the PA session, which may suggest a delayed therapeutic effect. This pattern may propose that participants may require time to process and internalize the insights gained from the PA before perceiving meaningful change. Effectively, while symptoms such as psychological distress and depression are typically tied to emotional states that can fluctuate quickly and are often readily accessible to self-report (Clark and Watson, 1991), the perception of psychological change reflects a more reflective, metacognitive process. It often requires self-narration and integration of new emotional and cognitive insights into one’s self-concept, a process that naturally unfolds over time (Wilson and Dunn, 2004; Greenberg, 2002). As such, changes in self-perception may occur more slowly than changes in emotional distress, emerging only after individuals have had time to reflect on their experiences and integrate the information gained during the PA.

In contrast, no improvement was observed in wellbeing. The null model was preferred, and Bayesian factors provided moderate to strong support for the absence of an effect of the FRAX-TA on this outcome. This finding may suggest that wellbeing, as measured by the CBA-VE, may reflect more stable, trait-like characteristics that are less sensitive to brief, time-limited interventions, such as a SS-CBT (Testa and Simonson, 1996; Diener et al., 1999). Wellbeing is a complex, multidimensional construct encompassing life satisfaction, meaning, and long-term emotional balance, and it typically changes gradually over time, often requiring ongoing therapeutic support or broader systemic interventions.

In addition, many female PCs were caregivers of children with FXS, and chronic caregiving stress, combined with the increased vulnerability to emotional dysregulation associated with PM status, may contribute to a more persistent reduction in wellbeing that is unlikely to be fully addressed through a single psychoeducational or therapeutic session (Bailey et al., 2008a,b; Wheeler et al., 2014a). However, previous analyses conducted on the same cohort comparing PCs who were mothers of children with FXS and PCs without children with FXS did not identify significant differences across the psychological variables assessed (Montanaro et al., 2026). These findings suggest that caregiving status alone may not fully account for the psychological patterns observed in this sample and that well-being may represent a domain requiring more sustained therapeutic engagement than can be achieved through a brief SSI.

In sum, while FRAX-TA provides meaningful psychological benefits, it should be considered an entry point rather than a substitute for ongoing care. Given the under-recognition of FXAND in clinical settings (Espinel et al., 2016; Budimirovic et al., 2018; Hagerman et al., 2018; Montanaro et al., 2024a), these findings carry important public health implications. FRAX-TA’s structured, relational structure may facilitate symptom recognition and emotional relief. However, to fully meet participants’ needs, it should ideally be complemented by more comprehensive and long-term psychological interventions (Sodhi and Hagerman, 2021).

*H2:* Middle-term effect of FRAX-TA process following PA

We hypothesized that PA would generate a distinct therapeutic benefit, producing the most pronounced improvement immediately after its delivery. This hypothesis received partial support from the data. Bayesian comparisons provided evidence of improvement in depression and psychological distress between the CBA-VE_pre and CBA-VE_afterPA, with these gains generally sustained at follow-ups (CBA-VE post). While this pattern may point to the potential effectiveness of the SS-CBT component in alleviating emotional burden, the findings should be interpreted with caution. Given the single-session format and the complexity of participants’ experiences, it remains possible that additional uncontrolled factors contributed to the observed changes.

In contrast, the effect on anxiety did not appear immediately after the PA session. Although no significant change was observed at that point, a significant improvement was evident by the follow-up phase, suggesting that reductions in anxiety may have emerged gradually over time. This delayed pattern is broadly consistent with findings from both SS-CBT and broader CBT literature, which indicate that while some cognitive and affective symptoms (e.g., dysfunctional thoughts, distress) may respond relatively quickly to psychoeducation and cognitive reframing, anxiety symptoms may decrease more gradually as individuals continue to reflect on and apply therapeutic insights in their daily lives (Hofmann et al., 2012; Kazantzis et al., 2018; Cohen et al., 2023; Papola et al., 2024). This pattern may be particularly relevant in populations characterized by chronic or genetically linked vulnerability, such as female PCs, who may require more time to process and integrate psychological information due to specific neurocognitive and emotional features (Wheeler et al., 2014b; Hessl et al., 2024). At the same time, these findings should be interpreted with caution. The absence of a control group and the modest magnitude of some effects limit the ability to attribute observed changes directly to the FRAX-TA intervention. Therefore, the present results should not be interpreted as evidence that a SSI can substitute for more comprehensive psychological treatment. Rather, they suggest that embedding a brief SS-CBT-informed session within an assessment context may offer a potentially useful complementary approach. Even in the absence of ongoing therapy, participants reported reductions in distress that may indicate early emotional adjustment following the session. While preliminary, these observations point to the potential value of a single, well-timed, and structured session in facilitating reflection and coping, particularly in contexts where access to longer-term therapy is limited or where assessment itself represents the primary psychological contact, such as in clinical trials (Stallard, 2020; Durosini and Aschieri, 2021).

Consistent with this interpretation, a notable strength of the PA session was its ability to fulfill its psychodiagnostic function, crucial in both clinical and research contexts, while simultaneously promoting self-validation and awareness. Many individuals may have difficulty distinguishing between normative psychological fluctuations and clinically significant symptoms. In this light, the PA session may have facilitated more accurate symptom recognition, enabling participants to better understand and articulate their experiences. As an illustration of this point, while only three participants self-identified as having FXAND prior to the study, the administration of the SCID-5 during the PA session revealed that 41 out of 81 participants met criteria for at least one lifetime SCID-5 condition. Although the cognitive and psychological characterization of participants was not the primary aim of this study, this finding is noteworthy, as standard questionnaires or medical records may underestimate the prevalence of these conditions (Montanaro et al., 2025). This pattern may also help contextualize the temporal divergence observed across outcomes. The immediate reduction in depressive symptoms and psychological distress may reflect the sense of relief and validation that can emerge when individuals are able to recognize and label their experiences more clearly. In contrast, the delayed reduction in anxiety may reflect the more gradual process of integrating this information and adjusting coping strategies in response to increased awareness. In the context of the PM, these processes may be particularly relevant. Many PCs remain unaware that their psychological symptoms could be related to their genetic status. As illustrated by one participant’s comment- “*If I had known I had FXAND, I would have done something earlier*”- structured feedback delivered within a respectful and educational framework may play an important role not only in reducing uncertainty but also in supporting informed decision-making and potentially facilitating earlier access to appropriate care.

*H3*: PA delivery week effect

Our final hypothesis examined whether the timing of the PA session would moderate psychological outcomes. We expected that earlier delivery might lead to more sustained gains by providing therapeutic input at a formative stage in the assessment process. The results, however, revealed a more complex and outcome-specific picture. For most core symptoms, including anxiety, depression, and psychological distress, timing effects were minimal or null. Bayesian model comparisons generally did not support meaningful interactions between intervention phase and timing. This suggests that the overall structure and content of the FRAX-TA protocol, rather than the precise week of intervention, accounted for most symptom improvement.

In contrast, a moderate timing-dependent effect emerged for the Perception of Positive Change. Specifically, participants in Group B, who received the PA session at week 6, demonstrated greater improvement in perceived change compared to those in Group C (PA at week 8), with moderate post-hoc evidence supporting this difference. Other between-group comparisons did not yield substantial support, indicating that any timing effect was neither linear nor consistent across all groups. Interestingly, although early administration of the PA (Group A, week 4) appeared to prompt a sharper initial increase in perceived change, this gain was not sustained over time (see Figure 2).

Taken together, these findings suggest that allowing a longer period for self-reflection and contextual processing prior to receiving feedback may promote more enduring psychological benefits. This pattern may reflect a psychological “window of readiness,” during which individuals may be more receptive to therapeutic input and better positioned to experience meaningful growth. A mid-phase delivery may offer participants time to build trust in the process, reflect on their functioning, and become emotionally prepared to receive and internalize therapeutic input. This interpretation aligns with existing therapeutic and developmental literature, which highlights the importance of timing in promoting insight, change readiness, and intervention efficacy (Finn, 2007; Tharinger et al., 2022).

Finally, consistent with prior findings, the CBA-VE wellbeing did not show significant changes across any timing condition. This offers support to the interpretation that trait-level wellbeing may be less responsive to brief or time-limited interventions. It is possible that wellbeing reflects more enduring psychological dimensions, such as life satisfaction and purpose, which may improve only indirectly, often following longer-term reductions in emotional distress or functional gains achieved through sustained intervention (Keyes, 2005; Ryff and Singer, 1998).

## 7 Clinical and research implications

The present findings provide preliminary support for the clinical utility of embedding a SS-CBT-informed intervention within an assessment-based protocol for PCs. Even in the absence of ongoing therapy, participants experienced reductions in emotional distress and depressive symptoms, suggesting that brief, structured interventions can offer initial meaningful support, mostly in clinical trials where often treatment is not the primary objective. Importantly, the timing of the PA session did not substantially change outcomes for core symptoms such as anxiety or depression, indicating that the therapeutic value of the FRAX-TA may be consistent across the different time-points. The long-term effect of FRAX-TA process suggests the adaptation of similar interventions in diverse clinical settings without the need for rigid scheduling. Nonetheless, the timing of PA delivery appeared to influence subjective perceptions of positive change, pointing that mid-phase intervention may offer an optimal window for cognitive-emotional integration. As anticipated, the study introduces a novel hypothesis that participants’ psychological readiness shaped by prior engagement, ongoing monitoring and relationship-building may influence both their symptom disclosure and their capacity to internalize the effects of a structured psychosocial intervention.

Women with the PM represent a genetically vulnerable, diagnostically complex, and often under-recognized population. Despite known risks for anxiety, depression, cognitive decline, and FXPAC-related conditions, most participants in this study had no prior psychological diagnosis or intervention experience. FRAX-TA thus served as both a clinical entry point and an educational tool, allowing participants to connect personal difficulties to a recognized neurogenetic framework. Importantly, many women reported that their symptoms had been previously dismissed or misunderstood, either due to a lack of knowledge among professionals or because of personal stigma. In this sample, FRAX-TA helped to bridge this gap by naming the experience, reducing shame, and normalizing psychological distress within the context of their genetic status. This would explain the pattern of the initial underreporting in this study: participants often provided scores within the non-clinical range on the CBA-VE yet disclosed a substantially greater number and severity of symptoms during the PA session. This discrepancy is consistent with previous literature showing that PCs frequently minimize or fail to recognize their emotional and cognitive challenges when responding to self-administered questionnaires (Roberts et al., 2009; Gossett et al., 2016). Such differences may reflect both limited symptom insight and the internalization of stigma, reinforcing the value of relationally oriented and PA models. Precisely for this reason, we chose not to analyse participants data according to diagnostic subtypes (e.g., FXAND, FXTAS, FXPOI), but rather to consider the group as a whole. First, the cross-sectional design of the study precludes predictions about future clinical trajectories, which may emerge over time. Second, and more importantly, the primary aim of the FRAX-TA protocol was not diagnostic classification per se, but rather to foster awareness, promote psychoeducation, and encourage participants to actively engage in preventive or supportive actions. From a research perspective, these findings emphasize the methodological importance of combining self-report and clinician-administered tools, particularly in underdiagnosed populations. Integrating psychoeducational strategies within assessment protocols may increase symptom disclosure and enhance both the accuracy of prevalence data and the readiness of participants to seek appropriate care.

## 8 Limitations, future directions, and conclusion

Before drawing conclusions, several limitations of the present study should be acknowledged. First, the absence of a control or comparison group limits the ability to draw firm causal conclusions regarding the psychological improvements observed over time. Without a control condition, it is not possible to determine whether these changes are attributable specifically to the FRAX-TA intervention, repeated assessment procedures, or broader participant engagement in the study process. Therefore, the findings should be interpreted as preliminary and future studies should incorporate waitlist or active control groups to more precisely isolate the effects of each component of the intervention. Second, the sample consisted exclusively of Italian White women, most of whom were mothers of children with FXS. This demographic and caregiving context may shape psychological experiences and coping processes, limiting the generalizability of the findings to more diverse populations, including individuals from different cultural backgrounds, men, or PCs without caregiving responsibilities. Third, although the sample size was relatively large for a pilot intervention study (*N* = 81), subgroup analyses, particularly those examining the timing of the psychoeducational assessment, may have been underpowered to detect subtle interactions. Recruitment was constrained by strict inclusion criteria and voluntary participation among individuals aware of their carrier status. Fourth, while the timing of the PA session appeared to influence psychological outcomes, potential moderating variables such as age, CGG repeat length, caregiving burden, and psychiatric history were not examined. Future studies could explore these factors to better understand individual differences in response to the intervention. Fifth, although the CBA-VE has strong psychometric properties, it is a self-report instrument and may be susceptible to underreporting or response bias. Notably, participants disclosed more psychological symptoms during clinician-administered SCID-5 interviews than in the questionnaires, suggesting that structured clinical interviews may capture aspects of psychological distress not fully reflected in self-report measures. Finally, qualitative feedback was collected through an anonymous questionnaire developed specifically for this study, which has not undergone formal validation. Although responses were overwhelmingly positive, response bias cannot be excluded, as participants who experienced greater benefit may have been more likely to complete the survey.

Despite these limitations, this study contributes to the broader field of psychological assessment and psychotherapy research by demonstrating that TA and SS-CBT can be effectively integrated. These two models are shown here to be mutually reinforcing. TA provides the narrative and emotional grounding, while SS-CBT introduces practical strategies for symptom management. Additionally implementing FRAX-TA also highlighted the importance of specialized training, not only in CBT techniques and assessment interpretation, but in relational sensitivity and genetic knowledge. Assessors were trained to maintain both clinical rigor and emotional presence, suggesting that brief interventions may require clinical skills also when embedded in research contexts. This may suggest that psychologists, genetic counselors, pediatricians, and neuropsychiatrists should attend interdisciplinary training programs to be prepared to recognize and validate the psychological needs of PCs, while collecting genetic, medical, genetic and neuropsychological data.

Overall, this study provides preliminary insights into the psychological characterization of PCs and the potential application of TA in neurogenetic conditions. Future studies should aim to replicate these findings in larger and more diverse populations, explore personalized timing strategies, and evaluate the longer-term clinical and functional outcomes of this integrative assessment model.

## Data Availability

The raw data supporting the conclusions of this article will be made available by the authors, without undue reservation.
