# Supplementary material for: Optimizing single-session CBT delivery in an 8-session longitudinal therapeutic assessment (FRAX-TA) for women with FMR1 Premutation
Source: Front Mol Neurosci. 2026 Apr 24;19:1718675. doi: 10.3389/fnmol.2026.1718675 (PMC13152860; doi:10.3389/fnmol.2026.1718675)
Supplement: Supplementary file 1 [file Data_Sheet_1.pdf]

Appendix A. Detailed Structure of the FRAX-TA Protocol

This appendix provides a detailed description of the FRAX-TA protocol and its procedural components, complementing the overview presented in the main Methods section.

The FRAX-TA protocol integrates principles from Therapeutic Assessment (TA) and Single-Session Cognitive Behavioral Therapy (SS-CBT) within a structured multi-session framework. The protocol was designed to combine standardized psychological assessment procedures with relational engagement, psychoeducation, and brief cognitive-behavioral strategies tailored to individuals carrying the *FMR1* premutation (PM).

The protocol was organized into the following key phases:

1. *Welcoming Phase (WP):*

The first contact with participants was always made directly by the CBT therapist via phone call, followed by a written summary sent through email. This two-step approach was adopted to foster a warm and clear initial connection while ensuring accessibility and transparency. In many medical or research contexts, the initial outreach is often delegated to third parties, which can result in the transmission of information that is unclear, overly technical, or lacking in empathy. By having the CBT therapist personally initiate the contact, the FRAX-TA model aimed to immediately establish a sense of relational safety and trust. The therapist highlighted that each meeting in the upcoming protocol was designed to gather information, to offer space, attention, and understanding of the participants' daily lives and personal challenges. The follow-up email, which reiterated the same information provided during the phone call, served both as a practical reminder, particularly important for individuals who may experience high daily stress due to caregiving responsibilities. Finally, the e-mail itself served as a direct point of contact with the therapist, offering participants a stable and accessible channel for clarification or support throughout the study and, if needed, even beyond its completion. Each WP session lasted approximately 15 minutes.

2. *Assessment Onboarding Phase (AOP)*

Following the completion of the WP, participants were contacted by one of two trained assessors, who sent via e-mail the the Google Form link and conducted the first administration of the CBA-VE through a semi-structured telephone interview. This approach was intentionally designed to provide emotional support while ensuring methodological rigor, as the standardized nature of the CBA-VE guarantees both consistency and reliability in assessment. During the call, participants were also informed about the scheduling of upcoming appointments. This structured communication aimed to address a common challenge in clinical settings, where clients often experience uncertainty regarding timelines and the roles of different professionals, factors that together can contribute to disengagement or early dropout. Each AOP lasted around 20 minutes.

3. *Cognitive Screening Phase (CSP)*

The CSP, conducted via Zoom, always began with a brief check-in to explore the participant's current psychological state and any significant life events in recent weeks, facilitated through the administration of the CBA-VE. This was followed by a clear and empathetic explanation of the rationale behind the cognitive assessment. Assessors were trained to explain that cognitive decline is a gradual process, with performance often beginning to decline in most people after their twenties or thirties (Salthouse, 2009). As such, the CSP was designed not only to gather baseline cognitive data, but also to potentially serve as a reference point for future longitudinal monitoring or, when relevant,

to support the early implementation of preventive strategies in collaboration with the participant's healthcare provider. In this study, the CSP included administration of the Raven Standard Progressive Matrices (Raven, 2008) and the Telephone-Montreal Cognitive Assessment (T-MoCA) (Katz et al., 2021; Wang et al., 2023). At the end of the session, assessors informed participants that the results would be reviewed and further discussed with the CBT therapist during the PA session. Each CSP lasted around 60 minutes.

4. *The Psychoeducational Assessment (PA)*

The PA, conducted by the CBT therapist, was the longest session, with a total duration of approximately 100-120 minutes. It always began with a brief reiteration of the study's goals, followed by a clear explanation of what the participant would be doing during the session. The focus was placed on the participant's psychological and emotional landscape, and discussions about any children (or relatives) with FXS were intentionally set aside unless directly relevant to the participant's own condition. For mothers of children with FXS, a brief space at the end of the session was reserved for addressing any specific questions the participant might have about FXS, ensuring that her concerns as a mother were acknowledged without diverting attention from the central goal of focusing on her personal well-being. At this point, the therapist informed the participant that the cognitive test results had not been reviewed in advance, as they would be examined together during the session. This approach was intended to preserve objectivity and to promote a collaborative interpretive process, fostering a shared understanding grounded in the participant's lived experience. The joint review of results took place during one of the key phases of the session, which included the following components:

- Collection of Clinical History

This stage involved gathering comprehensive background information through a semi-structured interview. Particular attention was given to the participant's personal experiences related to the PM. Key open-ended questions included:

1. *When did you first learn that you had the FMR1 premutation?*
2. *Where and how did you receive your diagnosis?*
3. *If you discovered you were a carrier after learning that you had a child with FXS, were you provided with appropriate information about the premutation at that time?*
4. *Could you give an example of all the medical conditions you have faced or are currently facing, even if you do not believe they are linked to the premutation?*

This question served a dual purpose: it supported data collection for the broader study on PM characterization, while also providing an opportunity for psychoeducational feedback when participants reported conditions (e.g., fibromyalgia) that are already documented in the literature as associated with the PM.

- Exploration of Personal Needs and Perspectives on Public Health Policies

This part focused on understanding the participant's personal needs as a woman with the PM, as well as her perspectives on broader health and policy issues. Open-ended questions included:

1. *What are your needs as a person living with the PM?*

2. *What actions should the State or Public Health authorities undertake to support individuals with the PM?*

3. *In your opinion, what services should be made available to individuals carrying the premutation?*

4. *Would you agree with the implementation of pre- or neonatal screening for the FXS?*

Regarding the last question, the CBT therapist discussed with each participant the potential advantages and disadvantages of pre- or neonatal screening. This dialogue included a shared reflection on some of the key ethical issues described in the scientific literature (Kaye, 2023; Tassone et al., 2023), ensuring that participants were informed and could critically engage with the topic.

The inclusion of this phase was considered essential within the framework of the PA and FRAX-TA process. First, exploring the participant's personal needs would promote a deeper understanding of her lived experience with the PM, potentially empowering her to articulate unmet needs and to reflect on possible avenues for personal and systemic support. Second, encouraging participants to express their views on public health initiatives would not only foster a sense of agency and validation but also help participants better recognize and clarify their own needs. Reflecting on what the State or Public Health system should provide could facilitate insight into personal areas requiring attention, potentially motivating participants to take specific actions, such as seeking out specialized medical care (e.g., consulting a gynaecologist familiar with PM-related conditions such as FXPOI). Third, discussing complex issues such as genetic screening and its ethical implications would provide participants with updated scientific and ethical knowledge, supporting critical thinking and emotional processing of sensitive topics. This integrative process was intended to bridge personal experiences with broader societal issues, reinforcing the psychoeducational component of the intervention and facilitating a collaborative and respectful exploration of identity, needs, and values.

- Psychodiagnostic Evaluation

The psychodiagnostic evaluation was conducted using the Structured Clinical Interview for DSM-5 Disorders (SCID-5) (First at al., 2015). Each evaluation began with a brief psychoeducational introduction, emphasizing that psychological difficulties are very common across the global population. To normalize the experience of psychological symptoms and reduce potential stigma or discomfort, the therapist would provide concrete examples, such as explaining that the global prevalence of anxiety disorders is estimated to be around 7% (Javaid et al., 2023) and that depressive disorders affect more than 5% of the world's population (World Health Organization, 2023). Participants were also informed that PM increases the risk of developing conditions associated with the FXAND spectrum. This phase was therefore important for two main reasons: first, acknowledging the presence of psychological difficulties could contribute to advancing scientific understanding of the prevalence and clinical features of FXAND; second, identifying potential issues would help inform appropriate therapeutic, preventive, or supportive actions for the participant herself. At the end of this part, participants were asked about current or past use of medication and counselling services.

- Feedback on CSP

At this stage, the therapist shared the participant's results on the Raven's Progressive Matrices and the T-MoCA. The therapist explained the meaning of the numerical results, helping the participant contextualize her performance and addressing any questions or emotional reactions that arose during the discussion.

- Consolidation and Empowerment Phase

In the final part of the session, the CBT therapist provided a collaborative summary of the key themes and insights that had emerged during the assessment. This recap aimed to reinforce the participant's understanding and to highlight her personal resources and areas for growth. In addition, the therapist introduced evidence-based Integrative Health Strategies for individuals carrying the PM, including recommendations related to nutrition, exercise, and lifestyle modifications. Participants were informed that a more detailed resource, drafted by the therapist, would subsequently be sent to them via e-mail in the form of an e-book (Appendix B). Finally, the PA session incorporated elements from third-wave CBT. These included psychoeducation about the interaction between thoughts, emotions, and behaviors, normalization of psychological responses associated with the premutation condition, and the identification of personally meaningful coping strategies. When clinically appropriate, the therapist used the ABC model (Activating event- Beliefs- Consequences; Hofmann et al., 2013) to help participants reflect on how specific interpretations of events could influence emotional reactions and behavioral responses. This process was used not as a structured cognitive restructuring protocol, but as a brief conceptual tool to facilitate insight and promote flexible responses to stressors. Consistent with third-wave CBT approaches (e.g., Acceptance and Commitment Therapy and related contextual behavioral models; Hayes, 2016; Wampold, 2015), the PA also emphasized values clarification, psychological flexibility, and acceptance of internal experiences, particularly in relation to the complex identity and health-related challenges associated with the PM. These strategies were integrated into the collaborative interpretation of assessment results to support participants in recognizing both vulnerabilities and strengths, while identifying adaptive ways of responding to distress.

- Administration of the CBA-VE

The session concluded with the administration of the CBA-VE to evaluate any immediate emotional and cognitive effects resulting from the PA experience.

5. *Phone CBA-VE*

Two weeks after the PA, assessors continued to contact each group by phone until the end of the study, for a total of eight time points. Each CBA-VE administration lasted approximately 15-20 minutes.

6. *Anonymous Feedback Online Questionnaire (optional)*

At the end of the study, participants were invited to complete an anonymous, semi-structured questionnaire administered via Google Forms.

References:

1. First M.B., Williams J.B.W., Karg R.S., Spitzer R.L. Structured Clinical Interview for DSM-5 Research Version. American Psychiatric Association; Washington, DC, USA: 2015
2. Hofmann, S. G., Asnaani, A., Vonk, I. J., Sawyer, A. T., & Fang, A. (2012). The Efficacy of Cognitive Behavioral Therapy: A Review of Meta-analyses. *Cognitive Therapy and Research*, 36(5), 427–440. Javaid, S.F., Hashim, I.J., Hashim, M.J. *et al.* Epidemiology of anxiety disorders: global burden and sociodemographic associations. *Middle East Curr Psychiatry* **30**, 44 (2023).
3. Javaid, S. F., Hashim, M. J., Stip, E., & Ahbabi, A. (2023). Epidemiology of anxiety disorders: global burden and sociodemographic associations. *Middle East Current Psychiatry*, *30*(1). https://doi.org/10.1186/s43045-023-00315-3
4. Katz, M. J., Wang, C., Nester, C. O., Derby, C. A., Zimmerman, M. E., Lipton, R. B., Sliwinski, M. J., & Rabin, L. A. (2021). T-MoCA: A valid phone screen for cognitive impairment in diverse community samples. *Alzheimer's & dementia (Amsterdam, Netherlands)*, *13*(1), e12144.
5. Kaye D. K. (2023). Addressing ethical issues related to prenatal diagnostic procedures. *Maternal health, neonatology and perinatology*, *9*(1), 1.
6. Raven, J. C. (2008). *SPM – Matrici Progressive Standard di Raven: Manuale di istruzioni (4^a edizione)*. Firenze: Giunti Psychometrics.x
7. Salthouse, T. A. (2009). When does age-related cognitive decline begin? *Neurobiology of Aging, 30*(4), 507–514. https://doi.org/10.1016/j.neurobiolaging.2008.09.023
8. Tassone, F., Protic, D., Allen, E. G., Archibald, A. D., Baud, A., Brown, T. W., Budimirovic, D. B., Cohen, J., Dufour, B., Eiges, R., Elvassore, N., Gabis, L. V., Grudzien, S. J., Hall, D. A., Hessl, D., Hogan, A., Hunter, J. E., Jin, P., Jiraanont, P., Klusek, J., … Hagerman, R. J. (2023). Insight and Recommendations for Fragile X-Premutation-Associated Conditions from the Fifth International Conference on *FMR1* Premutation. *Cells*, *12*(18), 2330.
9. Wampold B. E. (2015). How important are the common factors in psychotherapy? An update. *World psychiatry : official journal of the World Psychiatric Association (WPA)*, *14*(3), 270–277. https://doi.org/10.1002/wps.20238
10. Wang, C., Nester, C. O., Chang, K., Rabin, L. A., Ezzati, A., Lipton, R. B., & Katz, M. J. (2023). Tracking cognition with the T-MoCA in a racially/ethnically diverse older adult cohort. *Alzheimer's & dementia (Amsterdam, Netherlands)*, *15*(1), e12410.
11. World Health Organization. (2023). *Depression*. https://www.who.int/news-room/fact-sheets/detail/depression
