# Supplementary material for: Optimizing single-session CBT delivery in an 8-session longitudinal therapeutic assessment (FRAX-TA) for women with FMR1 Premutation
Source: Front Mol Neurosci. 2026 Apr 24;19:1718675. doi: 10.3389/fnmol.2026.1718675 (PMC13152860; doi:10.3389/fnmol.2026.1718675)
Supplement: Supplementary file 2 [file Data_Sheet_2.pdf]

INTEGRATIVE HEALTH STRATEGIES IN INDIVIDUALS CARRYING THE *FMR1* PREMUTATION

Dr. Alice Montanaro, Cognitive-Behavioral Therapist
alicemontanaropsicologa@gmail.com
dott.ssaalicemontanaro

THE SCOPE OF THIS GUIDE

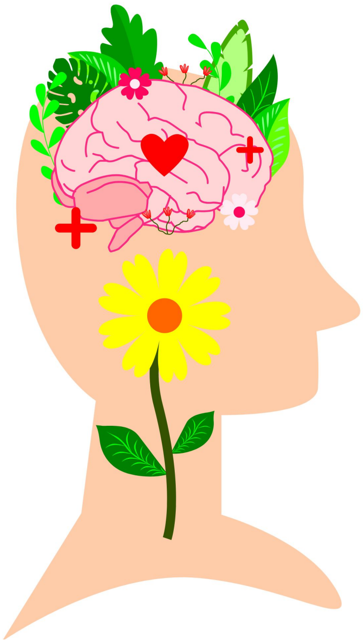

Welcome. My name is Dr. Alice Montanaro, and I am a Cognitive-Behavioral Therapist who has had the privilege of working with individuals carrying the *FMR1* premutation (PM) and Fragile X Syndrome (FXS) for several years. Through this experience, I have come to appreciate not only the scientific complexity of these conditions but also the difficulty that many face in accessing clear, practical guidance rooted in research. Although scientific understanding of the PM has advanced considerably over the past two decades, translating that knowledge into actionable, everyday strategies remains a significant hurdle.

I created this guide to bridge that gap: offering accessible, research-informed strategies to support both physical and emotional health. This guide is intended for individuals carrying the PM, their families, and anyone seeking to better understand how lifestyle factors can positively influence long-term outcomes. The goal is not to provide a one-size-fits-all solution, but rather to empower individuals with knowledge — giving them the tools to make informed, proactive choices for their well-being and resilience.

The guide was created for informational purposes, so before making important decisions about lifestyle changes (e.g., changes in diet, medications), it is recommended to consult your doctor.

INTRODUCTION

The PM involves an **expansion of CGG** repeats (55-200 CGGs) within the *FMR1* gene. While originally thought to be clinically silent, it is now recognized that carriers are at increased risk for a range of cognitive, psychiatric, and medical conditions grouped under the term Fragile X Premutation Associated Conditions (FXPAC) [Johnson et al., 2020]. At the biological level, the pathophysiology underlying FXPAC is attributed to RNA toxicity: elevated levels of *FMR1* mRNA interfere with normal cellular processes, leading to oxidative stress, mitochondrial dysfunction, calcium dysregulation, and ultimately enhanced neural cell death [Sodhi & Hagerman, 2021; Tassone et al., 2023].

FXPAC encompasses three main categories:

- **Fragile X-associated Primary Ovarian Insufficiency (FXPOI):** loss of normal function of the ovaries before the age of 40. **To note:** Some women with FXPOI may still conceive naturally, and hormonal fluctuations can continue unpredictably, unlike the permanent hormonal shutdown seen in natural menopause.
- **Fragile X-associated Tremor/Ataxia Syndrome (FXTAS):** a late-onset neurodegenerative disorder characterized by movement difficulties and cognitive decline, which can affect both men and women. **Notably,** in women, the clinical manifestation of FXTAS can be more subtle and difficult to depict; therefore, continuous monitoring is advised.
- **Fragile X-associated Neuropsychiatric Disorders (FXAND):** encompass a range of symptoms including anxiety, depression, attentional challenges, executive functioning difficulties, autistic traits, and emotional dysregulation, among others. Symptomatology can sometimes be difficult to identify due to stigma and under recognition; however, **it is important to acknowledge** that individuals with a diagnosis of FXAND may be at increased risk of developing FXTAS symptoms later in life. Early depiction is then essential for prevention!

Although not every carrier will develop clinical symptoms, recognizing these potential vulnerabilities is crucial for early intervention and prevention.

WHILE THERE IS CURRENTLY NO WAY TO COMPLETELY PREVENT OR REVERSE PREMUTATION-ASSOCIATED CONDITIONS, A COMBINATION OF TARGETED TREATMENTS AND LIFESTYLE MODIFICATIONS CAN HELP MANAGE SYMPTOMS EFFECTIVELY AND REDUCE THE RISK OF LONG-TERM COMPLICATIONS.

THIS GUIDE PROVIDES RESEARCH-INFORMED RECOMMENDATIONS — COVERING NUTRITION, PHYSICAL ACTIVITY, PSYCHOLOGICAL STRATEGIES, AND MEDICAL CONSIDERATIONS — DESIGNED TO SUPPORT SYMPTOM MANAGEMENT, STRENGTHEN RESILIENCE, AND ENHANCE QUALITY OF LIFE FOR INDIVIDUALS CARRYING THE PM.

NUTRITIONAL INTERVENTIONS

Oxidative stress and chronic inflammation are key mechanisms implicated in the pathology associated with the PM (Hagerman & Hagerman, 2018). Nutritional strategies aimed at reducing oxidative damage are therefore particularly relevant. Beyond targeting specific biological pathways, maintaining a balanced and nutrient-rich diet promotes both physical and psychological well-being in the short and long term.

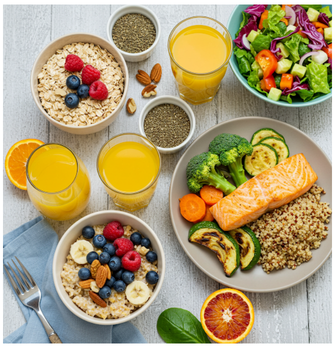

There is growing recognition of the bidirectional connection between the brain and the body, often referred to as the "gut-brain axis," which highlights how dietary habits can influence mood regulation, cognitive function, and overall mental health (Mayer et al., 2015; Foster et al., 2017). For individuals carrying the PM, who may be at higher risk of experiencing anxiety, depression, and cognitive difficulties—particularly if they are parents of children with FXS, which can add daily life challenges—adopting an **anti-inflammatory, antioxidant-rich diet** may help not only mitigate biological risk factors but also support emotional resilience and quality of life. A balanced intake of macronutrients, regular consumption of fruits and vegetables, and attention to gut health through prebiotics and probiotics could thus represent accessible and empowering strategies for self-care and prevention. Specific nutrients and bioactive compounds have been identified for their potential protective effects in this context.
The table below summarizes key examples:

ANTIOXIDANTS AND ANTI-INFLAMMATORY COMPOUNDS

Nutrient/Compound	Potential Benefits	Sources
**Sulforaphane**	Enhances antioxidant defenses; may reduce neuroinflammation	Broccoli sprouts, cruciferous vegetables
**Epigallocatechin gallate (EGCG)**	Promotes mitochondrial health; antioxidant properties	Green tea
**Omega-3 Fatty Acids**	Reduces inflammation; supports cognitive function	Fish oil, flaxseed, walnuts
**Vitamin E and C**	Antioxidant defense; protection against neurodegeneration	Nuts, seeds, citrus fruits
**Curcumin**	Anti-inflammatory and antioxidant properties; may support mood and cognitive function	Turmeric
**Magnesium**	Supports neurological function; may reduce	Leafy greens, almonds, avocados

	anxiety and promote relaxation	
**B Vitamins (B6, B9, B12)**	Essential for brain health and neurotransmitter synthesis; may help regulate mood and cognitive function	Whole grains, legumes, leafy greens, eggs
**Polyphenols (e.g., flavonoids)**	Reduce oxidative stress and inflammation; may protect cognitive function	Berries, dark chocolate, red grapes
**Coenzyme Q10 (CoQ10)**	Supports mitochondrial health and energy production; antioxidant effects	Organ meats, fatty fish, supplements

Incorporating these foods into daily meals can be a simple yet powerful step. For example, starting the day with a cup of green tea, adding a handful of walnuts to a salad, preparing a broccoli pizza for a fun and healthy meal with or without children, or regularly including steamed broccoli or other cruciferous vegetables can steadily build antioxidant capacity and reduce inflammation. Moreover, supporting gut health through prebiotics (such as fiber-rich foods) and probiotics (like fermented foods) may further enhance mental and physical well-being. Ultimately, small, consistent dietary changes—focusing on whole, minimally processed foods rich in antioxidants, healthy fats, and essential nutrients—offer accessible and empowering strategies for self-care and prevention in individuals carrying the PM.

Take Home Message

- Prioritize anti-inflammatory, antioxidant-rich foods.
- Daily choices build long-term resilience through gut and brain health.

EXERCISE RECOMMENDATIONS

PHYSICAL ACTIVITY IS WIDELY RECOGNIZED AS ONE OF THE MOST EFFECTIVE, EVIDENCE-BASED INTERVENTIONS FOR IMPROVING BRAIN HEALTH AND FOSTERING RESILIENCE ACROSS THE LIFESPAN. IT PLAYS A CRUCIAL ROLE IN MAINTAINING BOTH PHYSICAL AND MENTAL WELL-BEING, PARTICULARLY FOR INDIVIDUALS CARRYING THE **PM [SODHI & HAGERMAN, 2021].** REGULAR EXERCISE NOT ONLY CONTRIBUTES TO PHYSICAL HEALTH BUT ALSO HAS PROFOUND EFFECTS ON COGNITIVE FUNCTION AND EMOTIONAL STABILITY.

One of the key mechanisms through which exercise benefits the brain is its ability to boost levels of brain-derived neurotrophic factor (**BDNF**), a protein that promotes neuronal growth, repair, and plasticity. BDNF supports the formation of new neural connections and enhances the brain's capacity to adapt to new challenges, a process known as neuroplasticity. This is particularly

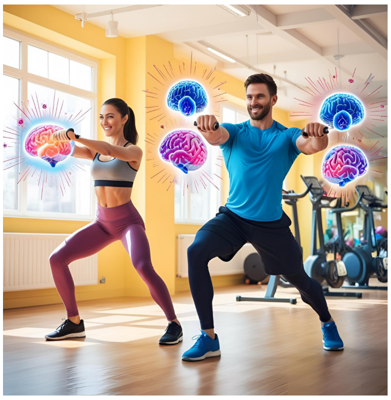

important for individuals with PM, as it may help mitigate some of the neurological challenges associated with the condition. Additionally, physical activity reduces systemic inflammation, which is linked to a variety of cognitive and mental health issues, and supports metabolic health—two critical factors for those carrying the PM.

Exercise has also been shown to reduce **mitochondrial dysfunction**, which is a key factor in aging, various neurodegenerative diseases and PM. Mitochondria are the powerhouses of cells, and their dysfunction can lead to decreased energy production and increased oxidative stress, both of which contribute to cognitive decline. By improving mitochondrial efficiency and enhancing cellular energy production, exercise helps support brain health and resilience. This combined effect of boosting BDNF, reducing inflammation, and improving mitochondrial function makes physical activity an essential strategy for individuals with PM, supporting both cognitive and metabolic health.

The table below summarizes key examples:

TYPES OF EXERCISE

Type of Exercise	*Benefits*	*Recommendations*	*Notes*
Aerobic Exercise	Improves cardiovascular health, enhances blood flow to the brain, boosts brain-derived neurotrophic factor (BDNF), reduces systemic inflammation, and improves mood.	Aim for at least 150 minutes per week of moderate-intensity activity, such as brisk walking, swimming, or cycling. Consider splitting the activity into 30-minute sessions, 5 days a week, or as per individual capabilities.	If needed, talk with your medical doctor before starting any new exercise routine.
Strength Training	Enhances metabolic health, increases muscle mass, improves bone density, boosts strength, and enhances stress resilience. It also helps in the prevention of sarcopenia (age-related muscle	Perform 2 sessions per week, targeting all major muscle groups. Include exercises like squats, lunges, push-ups, or weightlifting, aiming for 8-12 repetitions per set. Allow a day of rest between sessions for recovery.	Consult your doctor if you have any pre-existing health conditions.

	loss) and improves posture and stability.		
Mind-body Practices	Reduces anxiety, improves emotional regulation, enhances flexibility, and strengthens the connection between physical and mental health. Practices like yoga and tai chi improve mindfulness, reduce stress, and promote relaxation.	Engage in 1-2 sessions per week, lasting 30 to 60 minutes. Options include yoga, tai chi, or Pilates, focusing on breathwork, flexibility, and relaxation techniques.	If you are new to these practices or have any concerns, consider discussing with your healthcare provider first.

Take Home Message

- Exercise enhances cognitive, emotional, and physical resilience.
- Aim for a balance of aerobic, strength, and mind-body activities.

BEHAVIORAL THERAPY APPROACHES

Cognitive Behavioral Therapy (CBT) has shown efficacy in reducing anxiety and depressive symptoms in various genetic neurodevelopmental disorders and is promising for individuals with the PM (Hall et al., 2016). CBT focuses on identifying and **challenging negative thought** patterns and replacing them with more constructive, adaptive ways of thinking. It also emphasizes the development of healthier coping strategies for managing difficult emotions such as anxiety and depression.

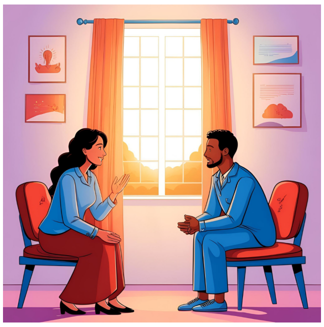

Research indicates that Cognitive Behavioral Therapy (CBT) can be highly effective in reducing symptoms of anxiety and depression (Zhang et al., 2019). By addressing the psychological challenges associated with genetic vulnerability, CBT helps individuals better understand and manage their emotional reactions to their genetic status, fostering improved emotional regulation and mental well-being. Furthermore, CBT can be integrated with parent training programs

for those raising children with Fragile X Syndrome (FXS). Anxiety and depression are widespread across the globe, and it's important to remember that these emotions are not something to blame yourself for—particularly when you have a genetic condition that may predispose you to certain symptoms.
It would be like feeling ashamed for being genetically predisposed to develop diabetes. Genetics are beyond our control, but we still have the power to take actions that can help us manage or reduce the risks associated with our genetic makeup. The same goes for emotional well-being—while we cannot control our genetic predispositions, we can take steps to address the psychological and emotional effects they may have.

Beneficial Behavioral Strategies for Emotional Resilience:

1. **Structured Problem-Solving for Daily Challenges:** Developing a structured approach to problem-solving can help individuals with genetic vulnerabilities manage the day-to-day challenges they face. By breaking down complex or overwhelming issues into manageable steps, problem-solving skills foster a sense of control and empowerment. This strategy is especially beneficial for tackling practical challenges, such as managing medical appointments, health screenings, or family planning decisions.

2. **Mindfulness-Based Stress Reduction (MBSR):** Mindfulness practices, including techniques such as meditation and focused breathing, are effective tools for reducing stress and improving emotional well-being. Mindfulness-Based Stress Reduction (MBSR) encourages individuals to live in the present moment, helping to reduce feelings of anxiety and worry about the future. By cultivating a mindful approach to life, individuals can experience improved emotional regulation and a sense of calm in the face of uncertainty.

3. **Psychoeducation to Normalize Emotional Responses:** Educating individuals about their genetic condition can help normalize the emotional responses that often arise from learning about one's genetic vulnerability. Psychoeducation provides essential information about the PM, including the potential risks, possible outcomes, and available interventions. By understanding the science behind their genetic status, individuals are better equipped to manage emotional reactions and make informed decisions about their health and well-being.

Take Home Message

- Emotional health is integral to overall well-being.
- Cognitive Behavioral Therapy (CBT), mindfulness, and education are key tools for fostering psychological resilience.
- **Don't feel ashamed for experiencing negative emotions**—they are a natural part of the human experience. Taking proactive steps to address and manage these emotions can help improve both mental and physical health.

COGNITIVE RESERVE AND COGNITIVE POTENTIATION

COGNITIVE RESERVE REFERS TO THE BRAIN'S ABILITY TO COMPENSATE FOR DAMAGE OR AGE-RELATED CHANGES THROUGH EFFICIENT NEURAL NETWORKS AND ALTERNATIVE COGNITIVE STRATEGIES. THIS CONCEPT IS PARTICULARLY RELEVANT FOR INDIVIDUALS CARRYING THE PM, WHO MAY BE AT INCREASED RISK OF COGNITIVE DECLINE DUE TO NEURODEGENERATIVE CHANGES OR VULNERABILITIES TO PSYCHOLOGICAL ISSUES.

WHAT CONTRIBUTES TO COGNITIVE RESERVE?

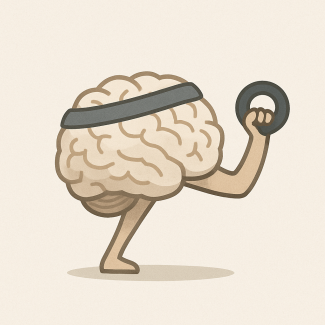

Studies have consistently shown that individuals with higher levels of **education**, **intellectual engagement**, and **lifelong learning** tend to have greater cognitive reserve (e.g., Stern et al., 2020). This means that even when their brains experience structural or metabolic stress (such as in neurodegenerative conditions like FXTAS), their cognitive performance remains more stable for longer.

Key contributors include:

- Formal education (e.g., university degrees or vocational training)
- Professionally or intellectually demanding occupations
- Bilingualism
- Active engagement in reading, puzzles, learning new skills
- Social stimulation and complex social environments

COGNITIVE POTENTIATION: BUILDING MENTAL STRENGTH

While cognitive reserve refers to a buffer built over time, **cognitive potentiation** refers to actively enhancing brain function in the present — much like "muscle-building" for the mind. This is especially important for individuals carrying the PM, as lifestyle-based potentiation may help delay or reduce the expression of cognitive symptoms.

The table below summarizes key examples:

Strategy Type	*Examples*	*Potential Benefits*
**Educational Enrichment**	Formal courses (e.g., languages, science, art), online learning platforms	Builds reserve; increases neural efficiency and compensatory capacity
**Cognitive Training**	BrainHQ, Cogmed, Lumosity; working memory or attention training programs	Enhances memory, processing speed, and attention

**Mind-Challenging Hobbies**	Chess, crossword puzzles, Sudoku, musical instruments, learning to code	Stimulates neuroplasticity; supports executive function
**Social Stimulation**	Group discussions, volunteering, teaching, mentoring	Supports theory of mind, verbal fluency, and emotional regulation
**Mindfulness & Meditation**	Guided meditation, focused attention training	Improves attentional control and reduces emotional reactivity
**Occupational Complexity**	Engaging in decision-making, problem-solving, or multitasking roles at work	Associated with higher resilience to age-related cognitive decline
**Cognitive Remediation**	Neuropsychologist-guided exercises (e.g., strategy-based learning)	Targets specific deficits; structured and evidence-based improvements

Take Home Message

- Building and maintaining **cognitive reserve** through education and mental activity provides long-term protection against cognitive decline.
- **Cognitive potentiation** involves actively challenging and stimulating the brain to enhance present-day functioning.
- It's **never too late** to start: cognitive benefits can be gained even in midlife or later.

PHARMACOLOGICAL OPTIONS

INDIVIDUALS WITH **THE PM** SHOULD ALSO CONSIDER EXPLORING MEDICATION OPTIONS AS PART OF THEIR **DAILY-LIFE**. CONSULTING WITH A MEDICAL DOCTOR WHO IS EXPERIENCED IN MANAGING CONDITIONS RELATED TO THE *FMR1* PREMUTATIONS CAN HELP ENSURE THAT MEDICATION CHOICES ARE TAILORED TO AN INDIVIDUAL'S SPECIFIC NEEDS AND GENETIC PROFILE.

The table below summarizes key pharmacological options explored by Sodhi and Hagerman [2021] for managing various symptoms associated with the PM and related conditions.:

Condition	Medication	Purpose/Benefits	Notes
**FXAND (Depression & Anxiety)**	SSRIs (e.g., sertraline, fluvoxamine, escitalopram)	Reduces symptoms of depression and anxiety.	Escitalopram and sertraline preferred for their favorable side effect profile and

			minimal drug interactions.
**FXAND (Depression & Anxiety)**	SNRIs (e.g., duloxetine, venlafaxine)	Treats both pain and depression/anxiety.	Recommended if pain symptoms or ADHD are present.
**FXAND (ADHD in Adults)**	SNRIs (e.g., duloxetine, venlafaxine)	Helps manage ADHD symptoms in adults with FXAND.	SNRIs can be more helpful for ADHD in adulthood.
**FXAND (Childhood ADHD)**	Stimulants (e.g., methylphenidate, dexamphetamine)	First-line treatment for ADHD.	Typically used after age 5 due to potential irritability at younger ages.
**FXAND (Childhood ADHD)**	Non-stimulants (e.g., atomoxetine, guanfacine)	Alternative treatments for ADHD if stimulants are not tolerated.	Guanfacine is helpful for overstimulated children and can be used under 5 years old.
**FXPOI (Hormone Replacement)**	Estrogen Replacement Therapy	Alleviates menopausal symptoms and helps manage osteoporosis.	Transdermal or transvaginal estrogen preferred due to lower thromboembolism risk.
**FXTAS (Movement Disorders)**	Propranolol, Primidone, Topiramate	Treats tremor and ataxia.	These medications help control movement symptoms in FXTAS.
**FXTAS (Movement Disorders)**	Riluzole, Amantadine, Buspirone, Sinemet	Additional options for managing ataxia and parkinsonian symptoms.	Sinemet (Parkinson's medication) is helpful if resting tremor or gait issues are present.
**FXTAS (Cognitive Dysfunction)**	Memantine	Improves cognitive function, especially in attention and memory.	Memantine has shown promise in improving cognitive function in FXTAS.
**FXTAS (Neuropathic Pain)**	Gabapentin, Pregabalin, Topical CBD	Treats neuropathic pain.	Gabapentin and pregabalin are commonly used for pain management.
**FXTAS (Neuroprotection)**	Allopregnanolone	Potential neuroprotective effects and may	Studied in open-label trials with positive results in

		improve cognitive deficits.	ERP and memory improvement.
**FXTAS (Psychiatric Issues)**	SSRIs (e.g., sertraline, fluvoxamine)	Treats psychiatric symptoms such as anxiety and depression.	SSRIs recommended for managing psychiatric symptoms in FXTAS.
**FXTAS (Cognitive & Motor Function)**	Citicoline	Potential benefits for motor and cognitive function.	Promising effects seen in trials, well tolerated in patients.

Take Home Message

- Pharmacological support can be valuable, but requires careful personalization.
- Collaborate with experienced clinicians for best outcome

CONCLUSION

Living with the PM may present unique challenges, but it also offers opportunities for proactive health management and self-empowerment. By making informed choices in nutrition, exercise, (neuro)psychological care, and, when necessary, medical management, individuals carrying the PM can take meaningful steps toward safeguarding their health and enhancing their overall quality of life. These choices help to build long-term resilience, fostering both physical and emotional well-being.

While research continues to evolve, one message is clear: small, consistent actions lead to powerful, positive change. This guide is designed to serve as a foundation for those actions, providing the knowledge and confidence needed to make decisions that support a full, vibrant life.

Embrace the opportunity to take control of your health, and remember that each step you take matters toward building a brighter future.

***Dr. Alice Montanaro*, Cognitive-Behavioral Therapist**
alicemontanaropsicologa@gmail.com
dott.ssaalicemontanaro

Acknowledgement

For further reading, please refer to the following scientific source:

Hagerman RJ and Hagerman PJ. Fragile X Syndrome and Premutation Disorders: New Developments and Treatments. London: Mac Keith Press; 2020.

Hagerman, R. J., Protic, D., Rajaratnam, A., Salcedo-Arellano, M. J., Aydin, E. Y., & Schneider, A. (2018). Fragile X-Associated Neuropsychiatric Disorders (FXAND). *Frontiers in psychiatry*, *9*, 564. https://doi.org/10.3389/fpsyt.2018.00564

Hunter, J. E., Jenkins, C. L., Grim, V., Leung, S., Charen, K. H., Hamilton, D. R., Allen, E. G., & Sherman, S. L. (2019). Feasibility of an app-based mindfulness intervention among women with an FMR1 premutation experiencing maternal stress. *Research in developmental disabilities*, *89*, 76–82. https://doi.org/10.1016/j.ridd.2019.03.008

Montanaro, F. A. M., Alfieri, P., Caciolo, C., Brunetti, A., Airoldi, A., de Florio, A., Tinella, L., Bosco, A., & Vicari, S. (2024). Fragile X Syndrome and FMR1 premutation: results from a survey on associated conditions and treatment priorities in Italy. *Orphanet journal of rare diseases*, *19*(1), 264. https://doi.org/10.1186/s13023-024-03272-0

Schneider, A., Summers, S., Tassone, F., Seritan, A., Hessl, D., Hagerman, P., & Hagerman, R. (2020). Women with Fragile X-associated Tremor/Ataxia Syndrome. Movement disorders clinical practice, 7(8), 910–919. https://doi.org/10.1002/mdc3.13084

Sodhi, D. K., & Hagerman, R. (2021). Fragile X Premutation: Medications, Therapy and Lifestyle Advice. *Pharmacogenomics and personalized medicine*, *14*, 1689–1699. https://doi.org/10.2147/PGPM.S338846

Tassanakijpanich, N., Hagerman, R. J., & Worachotekamjorn, J. (2021). Fragile X premutation and associated health conditions: A review. *Clinical genetics*, *99*(6), 751–760. https://doi.org/10.1111/cge.13924

Tassone, F., Protic, D., Allen, E. G., Archibald, A. D., Baud, A., Brown, T. W., Budimirovic, D. B., Cohen, J., Dufour, B., Eiges, R., Elvassore, N., Gabis, L. V., Grudzien, S. J., Hall, D. A., Hessl, D., Hogan, A., Hunter, J. E., Jin, P., Jiraanont, P., Klusek, J., … Hagerman, R. J. (2023). Insight and Recommendations for Fragile X-Premutation-Associated Conditions from the Fifth International Conference on *FMR1* Premutation. *Cells*, *12*(18), 2330. https://doi.org/10.3390/cells12182330

Walsh, M. B., Charen, K., Shubeck, L., McConkie-Rosell, A., Ali, N., Bellcross, C., & Sherman, S. L. (2021). Men with an FMR1 premutation and their health education needs. *Journal of genetic counseling*, *30*(4), 1156–1167. https://doi.org/10.1002/jgc4.1399

Johnson, K., Herring, J., & Richstein, J. (2020). Fragile X Premutation Associated Conditions (FXPAC). *Frontiers in pediatrics*, *8*, 266. https://doi.org/10.3389/fped.2020.00266
