# Supplementary material for: Optimizing single-session CBT delivery in an 8-session longitudinal therapeutic assessment (FRAX-TA) for women with FMR1 Premutation
Source: Front Mol Neurosci. 2026 Apr 24;19:1718675. doi: 10.3389/fnmol.2026.1718675 (PMC13152860; doi:10.3389/fnmol.2026.1718675)
Supplement: Supplementary file 3 [file Supplementary_file_1.docx]

Supplementary Material

**Table 1S.** Descriptive Measures distinguished by groups

|  | GROUP | Mean | SE | Med | SD | Min | Max |
| --- | --- | --- | --- | --- | --- | --- | --- |
| AGE | **A** | 50.5 | 2.50 | 49.5 | 11.74 | 31.7 | 81.0 |
|  | **B** | 51.2 | 2.33 | 47.9 | 10.68 | 35.7 | 68.8 |
|  | **C** | 51.2 | 2.13 | 48.0 | 9.02 | 39.9 | 61.8 |
|  | **D** | 49.2 | 1.10 | 49.5 | 4.94 | 40.9 | 81.0 |
| CGG REP | **A** | 94.0 | 3.80 | 88.5 | 17.84 | 63 | 131 |
|  | **B** | 83.4 | 4.01 | 83 | 18.39 | 58 | 126 |
|  | **C** | 87.8 | 4.54 | 82.5 | 19.26 | 57 | 150 |
|  | **D** | 97.1 | 7.24 | 86.5 | 32.40 | 57 | 199 |
| YEARS  EDUCATION | **A** | 14.5 | 0.699 | 13.0 | 3.28 | 8 | 20 |
|  | **B** | 15.0 | 0.751 | 16 | 3.44 | 8 | 20 |
|  | **C** | 15.5 | 0.887 | 13.0 | 3.76 | 8 | 22 |
|  | **D** | 15.3 | 0.646 | 16.0 | 2.89 | 8 | 20 |
| T0_ANX_T | **A** | 52.3 | 1.706 | 52.0 | 8.00 | 36 | 72 |
|  | **B** | 50.4 | 2.252 | 48 | 10.32 | 39 | 75 |
|  | **C** | 50.6 | 2.204 | 50.0 | 9.35 | 36 | 70 |
|  | **D** | 53.0 | 2.507 | 50.5 | 11.21 | 40 | 86 |
| T0_WELL_T | **A** | 51.0 | 2.053 | 49.5 | 9.63 | 38 | 78 |
|  | **B** | 52.0 | 2.241 | 55 | 10.27 | 25 | 72 |
|  | **C** | 50.4 | 1.966 | 47.5 | 8.34 | 39 | 73 |
|  | **D** | 48.1 | 1.694 | 49.0 | 7.58 | 34 | 60 |
| T0_POS_T | **A** | 51.0 | 2.053 | 49.5 | 9.63 | 38 | 78 |
|  | **B** | 51.5 | 2.206 | 53 | 10.11 | 25 | 72 |
|  | **C** | 50.4 | 1.966 | 47.5 | 8.34 | 39 | 73 |
|  | **D** | 48.1 | 1.694 | 49.0 | 7.58 | 34 | 60 |
| T0_DEPR_T | **A** | 57.6 | 2.157 | 56.5 | 10.12 | 43 | 80 |
|  | **B** | 54.0 | 2.293 | 51 | 10.51 | 42 | 80 |
|  | **C** | 56.4 | 2.475 | 53.5 | 10.50 | 43 | 77 |
|  | **D** | 59.1 | 2.638 | 56.5 | 11.80 | 44 | 92 |
| T0_DISTR_T | **A** | 57.8 | 2.192 | 56.5 | 10.28 | 43 | 80 |
|  | **B** | 53.8 | 2.331 | 51 | 10.68 | 41 | 80 |
|  | **C** | 56.5 | 2.503 | 53.5 | 10.62 | 43 | 78 |
|  | **D** | 59.9 | 2.820 | 56.5 | 12.61 | 44 | 95 |
| T1_ANX_T | **A** | 51.5 | 2.136 | 51.0 | 10.02 | 35 | 67 |
|  | **B** | 47.6 | 2.189 | 44 | 10.03 | 35 | 70 |
|  | **C** | 48.4 | 2.107 | 45.5 | 8.94 | 36 | 69 |
|  | **D** | 52.0 | 2.632 | 50.0 | 11.77 | 40 | 82 |
| T1_WELL_T | **A** | 48.2 | 2.318 | 46.0 | 10.87 | 34 | 80 |
|  | **B** | 53.9 | 1.998 | 55 | 9.16 | 32 | 68 |
|  | **C** | 48.9 | 1.717 | 48.5 | 7.28 | 31 | 59 |
|  | **D** | 45.1 | 1.474 | 45.0 | 6.59 | 34 | 58 |
| T1_POS_T | **A** | 47.5 | 1.758 | 48.0 | 8.24 | 32 | 63 |
|  | **B** | 51.4 | 2.149 | 49 | 9.85 | 35 | 75 |
|  | **C** | 50.6 | 1.536 | 51.5 | 6.52 | 35 | 59 |
|  | **D** | 46.6 | 1.982 | 45.0 | 8.86 | 35 | 66 |
| T1_DEPR_T | **A** | 57.1 | 2.310 | 55.0 | 10.84 | 42 | 75 |
|  | **B** | 51.2 | 1.532 | 50 | 7.02 | 42 | 65 |
|  | **C** | 53.7 | 1.773 | 54.0 | 7.52 | 45 | 75 |
|  | **D** | 58.5 | 2.408 | 54.0 | 10.77 | 46 | 85 |
| T1_DISTR_T | **A** | 52.8 | 2.778 | 47.5 | 13.03 | 38 | 80 |
|  | **B** | 46.0 | 2.123 | 44 | 9.73 | 37 | 80 |
|  | **C** | 49.2 | 2.384 | 45.5 | 10.11 | 40 | 75 |
|  | **D** | 51.4 | 2.277 | 50.0 | 10.18 | 38 | 75 |
| T2_ANX_T | **A** | 50.2 | 2.439 | 48.0 | 11.44 | 36 | 68 |
|  | **B** | 47.7 | 2.066 | 44 | 9.47 | 36 | 71 |
|  | **C** | 43.7 | 1.313 | 43.0 | 5.57 | 36 | 55 |
|  | **D** | 50.0 | 2.089 | 51.5 | 9.34 | 34 | 68 |
| T2_WELL_T | **A** | 53.0 | 3.008 | 50.0 | 14.11 | 27 | 82 |
|  | **B** | 52.9 | 1.900 | 53 | 8.71 | 39 | 71 |
|  | **C** | 50.4 | 2.411 | 49.5 | 10.23 | 32 | 73 |
|  | **D** | 45.3 | 1.897 | 47.5 | 8.48 | 28 | 60 |
| T2_POS_T | **A** | 52.3 | 2.593 | 51.0 | 12.16 | 29 | 74 |
|  | **B** | 50.9 | 1.810 | 51 | 8.30 | 38 | 73 |
|  | **C** | 47.3 | 1.878 | 46.0 | 7.97 | 30 | 68 |
|  | **D** | 48.4 | 2.281 | 47.0 | 10.20 | 26 | 63 |
| T2_DEPR_T | **A** | 53.6 | 2.422 | 50.0 | 11.36 | 42 | 75 |
|  | **B** | 51.1 | 1.797 | 48 | 8.24 | 41 | 69 |
|  | **C** | 50.1 | 1.263 | 49.0 | 5.36 | 43 | 63 |
|  | **D** | 55.4 | 1.605 | 55.0 | 7.18 | 45 | 72 |
| T2_DISTR_T | **A** | 46.1 | 1.928 | 43.5 | 9.04 | 36 | 67 |
|  | **B** | 44.2 | 1.251 | 42 | 5.73 | 37 | 58 |
|  | **C** | 44.1 | 1.523 | 41.0 | 6.46 | 37 | 61 |
|  | **D** | 46.2 | 1.625 | 45.0 | 7.27 | 38 | 67 |
| T3_ANX_T | **A** | 48.7 | 1.901 | 49.5 | 8.92 | 35 | 66 |
|  | **B** | 50.8 | 2.370 | 49.0 | 10.60 | 35 | 70 |
|  | **C** | 44.1 | 1.523 | 41.0 | 6.46 | 37 | 61 |
|  | **D** | 46.2 | 1.625 | 45.0 | 7.27 | 38 | 67 |
| T3_WELL_T | **A** | 48.2 | 2.390 | 48.0 | 11.21 | 26 | 77 |
|  | **B** | 54.8 | 2.591 | 56.5 | 11.59 | 32 | 73 |
|  | **C** | 51.6 | 2.514 | 51.0 | 10.67 | 34 | 71 |
|  | **D** | 47.7 | 1.920 | 49.5 | 8.58 | 30 | 62 |
| T3_POS_T | **A** | 50.5 | 2.228 | 49.0 | 10.45 | 36 | 77 |
|  | **B** | 47.1 | 1.760 | 45.5 | 7.87 | 32 | 64 |
|  | **C** | 48.6 | 2.148 | 48.0 | 9.12 | 26 | 64 |
|  | **D** | 47.5 | 2.001 | 46.0 | 8.95 | 30 | 70 |
| T3_DEPR_T | **A** | 53.4 | 1.781 | 54.0 | 8.35 | 40 | 69 |
|  | **B** | 49.7 | 1.203 | 50.0 | 5.38 | 41 | 61 |
|  | **C** | 51.1 | 1.490 | 52.0 | 6.32 | 40 | 63 |
|  | **D** | 55.3 | 2.031 | 54.0 | 9.08 | 42 | 76 |
| T3_DISTR_T | **A** | 45.7 | 1.633 | 43.5 | 7.66 | 36 | 66 |
|  | **B** | 41.9 | 0.971 | 40.0 | 4.34 | 37 | 55 |
|  | **C** | 43.8 | 1.366 | 41.5 | 5.80 | 38 | 57 |
|  | **D** | 47.1 | 1.771 | 45.0 | 7.92 | 38 | 64 |
| T4_ANX_T | **A** | 45.7 | 1.853 | 44.0 | 8.69 | 35 | 64 |
|  | **B** | 44.7 | 1.963 | 42.5 | 8.78 | 35 | 69 |
|  | **C** | 45.8 | 1.463 | 46.0 | 6.21 | 36 | 58 |
|  | **D** | 47.9 | 2.507 | 45.5 | 11.21 | 34 | 70 |
| T4_WELL_T | **A** | 48.2 | 2.568 | 49.0 | 12.05 | 26 | 84 |
|  | **B** | 55.5 | 2.018 | 56.0 | 9.02 | 34 | 71 |
|  | **C** | 49.7 | 2.250 | 47.0 | 9.55 | 32 | 67 |
|  | **D** | 50.9 | 2.578 | 54.0 | 11.53 | 25 | 66 |
| T4_POS_T | **A** | 48.5 | 1.948 | 47.0 | 9.14 | 34 | 72 |
|  | **B** | 53.5 | 1.754 | 53.5 | 7.84 | 39 | 67 |
|  | **C** | 42.3 | 2.305 | 40.0 | 9.78 | 28 | 63 |
|  | **D** | 47.7 | 2.460 | 46.0 | 11.00 | 30 | 68 |
| T4_DEPR_T | **A** | 51.0 | 1.952 | 50.0 | 9.15 | 41 | 70 |
|  | **B** | 50.8 | 1.850 | 49.0 | 8.28 | 40 | 76 |
|  | **C** | 50.3 | 1.207 | 50.0 | 5.12 | 42 | 65 |
|  | **D** | 53.6 | 2.429 | 51.5 | 10.86 | 40 | 77 |
| T4_DISTR_T | **A** | 44.3 | 1.819 | 40.0 | 8.53 | 36 | 72 |
|  | **B** | 43.9 | 1.788 | 42.0 | 8.00 | 36 | 69 |
|  | **C** | 40.9 | 0.716 | 40.5 | 3.04 | 37 | 46 |
|  | **D** | 47.4 | 1.939 | 43.5 | 8.67 | 37 | 66 |
| T5_ANX_T | **A** | 44.9 | 1.619 | 44.5 | 7.59 | 35 | 61 |
|  | **B** | 45.5 | 2.387 | 42.0 | 10.67 | 33 | 71 |
|  | **C** | 45.3 | 1.226 | 45.5 | 5.20 | 37 | 58 |
|  | **D** | 47.0 | 1.704 | 44.5 | 7.62 | 34 | 65 |
| T5_WELL_T | **A** | 48.8 | 1.831 | 48.0 | 8.59 | 36 | 75 |
|  | **B** | 54.9 | 2.338 | 57.0 | 10.46 | 36 | 79 |
|  | **C** | 49.7 | 1.858 | 50.5 | 7.88 | 33 | 62 |
|  | **D** | 51.0 | 1.461 | 50.0 | 6.53 | 40 | 62 |
| T5_POS_T | **A** | 49.0 | 1.960 | 48.0 | 9.19 | 29 | 71 |
|  | **B** | 53.3 | 1.992 | 52.0 | 8.91 | 38 | 79 |
|  | **C** | 49.4 | 1.981 | 49.0 | 8.40 | 36 | 64 |
|  | **D** | 45.4 | 1.618 | 45.5 | 7.23 | 33 | 59 |
| T5_DEPR_T | **A** | 49.8 | 1.433 | 48.5 | 6.72 | 42 | 66 |
|  | **B** | 50.1 | 2.110 | 47.5 | 9.44 | 40 | 78 |
|  | **C** | 50.9 | 1.703 | 50.5 | 7.23 | 42 | 71 |
|  | **D** | 50.2 | 1.241 | 49.0 | 5.55 | 41 | 62 |
| T5_DISTR_T | **A** | 43.1 | 1.552 | 40.0 | 7.28 | 36 | 63 |
|  | **B** | 43.6 | 1.850 | 40.5 | 8.27 | 36 | 69 |
|  | **C** | 44.6 | 1.599 | 43.5 | 6.78 | 36 | 56 |
|  | **D** | 42.4 | 0.887 | 41.5 | 3.97 | 37 | 50 |
| T6_ANX_T | **A** | 47.2 | 1.914 | 46.5 | 8.98 | 33 | 64 |
|  | **B** | 47.4 | 2.068 | 45.5 | 9.25 | 35 | 68 |
|  | **C** | 44.3 | 1.288 | 44.0 | 5.47 | 35 | 55 |
|  | **D** | 46.6 | 1.761 | 45.0 | 7.88 | 39 | 63 |
| T6_WELL_T | **A** | 46.1 | 2.389 | 43.0 | 11.21 | 31 | 75 |
|  | **B** | 54.1 | 2.301 | 53.0 | 10.29 | 40 | 85 |
|  | **C** | 49.5 | 2.000 | 50.5 | 8.49 | 31 | 69 |
|  | **D** | 49.5 | 2.181 | 49.5 | 9.75 | 27 | 68 |
| T6_POS_T | **A** | 47.9 | 2.208 | 47.0 | 10.36 | 26 | 71 |
|  | **B** | 54.0 | 1.904 | 52.0 | 8.52 | 42 | 80 |
|  | **C** | 48.1 | 2.010 | 48.0 | 8.53 | 32 | 66 |
|  | **D** | 48.3 | 2.230 | 47.0 | 9.97 | 33 | 68 |
| T6_DEPR_T | **A** | 51.5 | 1.663 | 49.0 | 7.80 | 40 | 67 |
|  | **B** | 50.0 | 1.871 | 47.5 | 8.37 | 40 | 74 |
|  | **C** | 50.5 | 1.365 | 49.5 | 5.79 | 40 | 61 |
|  | **D** | 52.8 | 1.992 | 52.0 | 8.91 | 41 | 74 |
| T6_DISTR_T | **A** | 45.6 | 1.730 | 44.0 | 8.12 | 37 | 72 |
|  | **B** | 44.0 | 1.787 | 43.5 | 7.99 | 36 | 66 |
|  | **C** | 43.7 | 1.484 | 41.5 | 6.29 | 36 | 58 |
|  | **D** | 45.1 | 1.680 | 42.0 | 7.51 | 38 | 63 |
| T7_ANX_T | **A** | 46.7 | 1.747 | 46.5 | 8.20 | 36 | 62 |
|  | **B** | 45.8 | 2.152 | 43.0 | 9.62 | 33 | 67 |
|  | **C** | 44.7 | 1.311 | 44.5 | 5.56 | 35 | 54 |
|  | **D** | 45.5 | 2.385 | 41.0 | 10.66 | 35 | 70 |
| T8_WELL_T | **A** | 46.9 | 2.459 | 46.0 | 11.54 | 32 | 80 |
|  | **B** | 53.5 | 1.963 | 54.0 | 8.78 | 34 | 73 |
|  | **C** | 46.6 | 2.447 | 46.5 | 10.38 | 30 | 73 |
|  | **D** | 49.6 | 2.019 | 53.0 | 9.03 | 28 | 58 |
| T7_POS_T | **A** | 49.4 | 2.395 | 47.0 | 11.23 | 30 | 78 |
|  | **B** | 54.1 | 1.653 | 56.0 | 7.39 | 42 | 66 |
|  | **C** | 46.2 | 1.840 | 46.0 | 7.81 | 32 | 61 |
|  | **D** | 48.3 | 1.892 | 50.0 | 8.46 | 35 | 63 |
| T7_DEPR_T | **A** | 51.3 | 1.833 | 51.0 | 8.60 | 40 | 70 |
|  | **B** | 50.3 | 1.918 | 46.5 | 8.58 | 40 | 68 |
|  | **C** | 49.7 | 1.563 | 48.5 | 6.63 | 39 | 61 |
|  | **D** | 53.1 | 2.385 | 49.5 | 10.67 | 42 | 76 |
| T7_DISTR_T | **A** | 45.1 | 1.954 | 42.0 | 9.17 | 36 | 72 |
|  | **B** | 42.5 | 1.612 | 40.0 | 7.21 | 36 | 62 |
|  | **C** | 43.6 | 1.324 | 41.0 | 5.62 | 36 | 55 |
|  | **D** | 45.5 | 2.319 | 40.5 | 10.37 | 37 | 72 |

*Legend.* Anx=CBA-VE anxiety; Well= CBA-VE wellbeing; Pos=CBA-VE positive changes; Depr= CBA-VE depression; Distr= CBA-VE Psychological distress;; M=mean; SE= standard error; Med=median; SD=standard deviation; Min= minimum score; Max= maximum score; Yrs=years; CGG=citosine-guanine-guanine repeats.

**Table 2S.** Baseline Demographic and Socioeconomic Characteristics Across Experimental Groups: Absence of Significant Differences

|  | | | | |
| --- | --- | --- | --- | --- |
| One Way Anova | **F** | **Df1** | **Df2** | **p** |
| Age | 0.397 | 3 | 39.9 | 0.756 |
| CGG REP | 1.602 | 3 | 41.5 | 0.203 |
| Yrs Education | 0.352 | 3 | 42.0 | 0.788 |
| χ² Test | **χ²** | **Df** |  | p |
| Income | 14.7 | 18 | | 0.681 |
| Marital Status | 20.7 | 12 | | 0.054 |

**Table 3S**. Descriptives (Mean, standard deviation, and total number) of the *Anxiety* score across the intervention phases for each group.

| **Phase of FRAX-TA** | | **Group** | | **Mean** | | **SD** | | **N** | |
| --- | --- | --- | --- | --- | --- | --- | --- | --- | --- |
| CBA-VE_PRE |  | A |  | 51.9 |  | 8.44 |  | 22 |  |
|  |  | B |  | 48.9 |  | 9.26 |  | 20 |  |
|  |  | C |  | 46.6 |  | 6.58 |  | 18 |  |
|  |  | D |  | 50.6 |  | 9.44 |  | 20 |  |
| CBA-VE_afterPA |  | A |  | 50.2 |  | 11.44 |  | 22 |  |
|  |  | B |  | 50.8 |  | 10.60 |  | 20 |  |
|  |  | C |  | 45.8 |  | 6.21 |  | 18 |  |
|  |  | D |  | 47.0 |  | 7.62 |  | 20 |  |
| CBA-VE_POST |  | A |  | 46.6 |  | 7.52 |  | 22 |  |
|  |  | B |  | 45.9 |  | 9.19 |  | 20 |  |
|  |  | C |  | 44.8 |  | 5.02 |  | 18 |  |
|  |  | D |  | 46.1 |  | 8.65 |  | 20 |  |
|  | | | | | | | | | |

**Table 4S.** Descriptives (Mean, standard deviation, and total number) of the *Depression* score across the intervention phases for each group.

| **Phase of FRAX-TA** | | **Group** | | **Mean** | | **SD** | | **N** | |
| --- | --- | --- | --- | --- | --- | --- | --- | --- | --- |
| CBA-VE_PRE |  | A |  | 57.3 |  | 10.00 |  | 22 |  |
|  |  | B |  | 52.4 |  | 8.18 |  | 20 |  |
|  |  | C |  | 52.8 |  | 6.77 |  | 18 |  |
|  |  | D |  | 56.4 |  | 9.26 |  | 20 |  |
| CBA-VE_afterPA |  | A |  | 53.6 |  | 11.36 |  | 22 |  |
|  |  | B |  | 49.7 |  | 5.38 |  | 20 |  |
|  |  | C |  | 50.3 |  | 5.12 |  | 18 |  |
|  |  | D |  | 50.2 |  | 5.55 |  | 20 |  |
| CBA-VE_POST |  | A |  | 51.4 |  | 7.27 |  | 22 |  |
|  |  | B |  | 50.3 |  | 7.91 |  | 20 |  |
|  |  | C |  | 50.4 |  | 6.22 |  | 18 |  |
|  |  | D |  | 52.9 |  | 9.13 |  | 20 |  |
|  | | | | | | | | | |

**Table 5S.** Descriptives (Mean, standard deviation, and total number) of the *Psychological Distress* score across the intervention phases for each group.

| **Phase of FRAX-TA** | | **Group** | | **Mean** | | **SD** | | **N** | |
| --- | --- | --- | --- | --- | --- | --- | --- | --- | --- |
| CBA-VE_PRE |  | A |  | 55.3 |  | 10.81 |  | 22 |  |
|  |  | B |  | 48.3 |  | 8.18 |  | 20 |  |
|  |  | C |  | 48.4 |  | 7.43 |  | 18 |  |
|  |  | D |  | 50.4 |  | 8.47 |  | 20 |  |
| CBA-VE_afterPA |  | A |  | 46.1 |  | 9.04 |  | 22 |  |
|  |  | B |  | 41.9 |  | 4.34 |  | 20 |  |
|  |  | C |  | 40.9 |  | 3.04 |  | 18 |  |
|  |  | D |  | 42.4 |  | 3.97 |  | 20 |  |
| CBA-VE_POST |  | A |  | 44.8 |  | 7.50 |  | 22 |  |
|  |  | B |  | 43.5 |  | 7.11 |  | 20 |  |
|  |  | C |  | 44.0 |  | 5.91 |  | 18 |  |
|  |  | D |  | 45.4 |  | 8.78 |  | 20 |  |
|  | | | | | | | | | |

**Table 6S.** Descriptives (Mean, standard deviation, and total number) of the *Positive Perception of Change* score across the intervention phases for each group.

| **Phase of FRAX-TA** | | **Group** | | **Mean** | | **SD** | | **N** | |
| --- | --- | --- | --- | --- | --- | --- | --- | --- | --- |
| CBA-VE_PRE |  | A |  | 46.3 |  | 6.46 |  | 22 |  |
|  |  | B |  | 48.7 |  | 5.76 |  | 20 |  |
|  |  | C |  | 47.5 |  | 4.71 |  | 18 |  |
|  |  | D |  | 47.1 |  | 6.35 |  | 20 |  |
| CBA-VE_afterPA |  | A |  | 52.3 |  | 12.16 |  | 22 |  |
|  |  | B |  | 47.1 |  | 7.87 |  | 20 |  |
|  |  | C |  | 42.3 |  | 9.78 |  | 18 |  |
|  |  | D |  | 45.4 |  | 7.23 |  | 20 |  |
| CBA-VE_POST |  | A |  | 49.1 |  | 9.58 |  | 22 |  |
|  |  | B |  | 53.7 |  | 7.42 |  | 20 |  |
|  |  | C |  | 47.9 |  | 7.88 |  | 18 |  |
|  |  | D |  | 48.3 |  | 8.93 |  | 20 |  |
|  | | | | | | | | | |

**Table 7S.** Descriptives (Mean, standard deviation, and total number) of the *Well-being* score across the intervention phases for each group.

| **Phase of FRAX-TA** | | **Group** | | **Mean** | | **SD** | | **N** | |
| --- | --- | --- | --- | --- | --- | --- | --- | --- | --- |
| CBA-VE_PRE |  | A |  | 49.6 |  | 9.65 |  | 22 |  |
|  |  | B |  | 52.9 |  | 8.93 |  | 20 |  |
|  |  | C |  | 50.3 |  | 8.26 |  | 18 |  |
|  |  | D |  | 47.4 |  | 7.45 |  | 20 |  |
| CBA-VE_afterPA |  | A |  | 53.0 |  | 14.11 |  | 22 |  |
|  |  | B |  | 54.8 |  | 11.59 |  | 20 |  |
|  |  | C |  | 49.7 |  | 9.55 |  | 18 |  |
|  |  | D |  | 51.0 |  | 6.53 |  | 20 |  |
| CBA-VE_POST |  | A |  | 47.6 |  | 9.92 |  | 22 |  |
|  |  | B |  | 54.5 |  | 8.28 |  | 20 |  |
|  |  | C |  | 48.6 |  | 8.35 |  | 18 |  |
|  |  | D |  | 49.6 |  | 8.73 |  | 20 |  |
|  | | | | | | | | | |

# Anonymous Feedback Questionnaire

1. Are you satisfied with your overall experience as a participant in this research project?

- ☐ Extremely
- ☐ Very
- ☐ Moderately
- ☐ Slightly

1. Did you feel supported and respected during your participation?

- ☐ Extremely
- ☐ Very
- ☐ Moderately
- ☐ Slightly

1. Has participating in the research helped you in any area of your life?

- ☐ Extremely
- ☐ Very
- ☐ Moderately
- ☐ Slightly

1. Are you satisfied with the overall experience you had?

- ☐ Extremely
- ☐ Very
- ☐ Moderately
- ☐ Slightly

1. Are you satisfied with the psychodiagnostic, and psychoeducational (PA) session conducted by the CBT therapist?

- ☐ Extremely
- ☐ Very
- ☐ Moderately
- ☐ Slightly

1. 6. Are you satisfied with your experience with Assessor A / Assessor B?

- ☐ Extremely
- ☐ Very
- ☐ Moderately
- ☐ Slightly

1. Would you like this research project on the premutation to continue in the coming years?

- ☐ Extremely
- ☐ Very
- ☐ Moderately
- ☐ Slightly

1. Open-ended question: In what area(s) of your life, if any, was the session with the CBT therapist supportive?

1. Open-ended question: In what area(s) of your life, if any, was the session with Assessor A/Assessor B supportive?

1. Open-ended question: Please feel free to share any general comments about the research you participated in or your personal reflections.

1. Open-ended question: Suggestions for the future

**Results**

The following pie charts illustrate the distribution of responses to the closed-ended questions included in the feedback questionnaire. Percentages are calculated based on a total of 62 participants.

Below are some of the most salient responses to the open-ended questions included in the questionnaire.

**Question 8:**

- Trying to find even a little bit of time during the day to dedicate to myself
- It made me aware of my situation and the relationship with my children
- In dealing with family dynamics and possible issues, especially regarding the sibling
- It highlighted the lack of importance I give to myself. I set aside my physical well-being to meet my children’s needs. Dedicating more time to myself, including through sports or physical activity
- I received some guidelines necessary for my path as a premutation carrier
- Awareness of having someone who takes care of the condition we live with
- Self-awareness, Support and encouragement, Anxiety management
- Strategies to cope with anxiety
- Strategies to help my children with FXS
- Knowledge
- Self-awareness

**Question 9:**

- It helped me accept my own particularities
- I had the session during a particularly anxious time in my life. I felt supported
- Excellent human and psychological support
- It helped me reflect on my emotions and how much they affect my life
- Very punctual and professional, while also empathetic and available
- By helping me recognize how I approach different situations, and by asking questions in a consistently discreet and respectful manner
- In the behavioral domain, making me reflect on certain personality traits

**Question 10**

- It was a way to begin gaining awareness of a condition that exists but is rarely discussed.
- I found it very important to draw attention to a genetic condition that is still little known, such as the FMR1 premutation. I hope that in the future, research can be further expanded and that, as a result, possible interventions can be identified to address the difficulties associated with the condition.
- It was interesting, although I believe that face-to-face contact is very helpful, so I hope there will be an opportunity in the future to give this research the recognition it deserves and that sufficient funding will be allocated to better understand this condition.
- I hope that research, both on the syndrome and the premutation, continues. Families—and especially women and mothers with the premutation—need ongoing support. I hope we will get to the point of thoroughly investigating the condition of the woman, or even better, of the couple before having children, so they are at least somewhat prepared for such a passionate, intense—and often uphill—life. Thanks to these wonderful researchers and their truly deserving work.
- Research, which is the foundation of all knowledge and progress, is in my view a fundamental and irreplaceable path.
  It can help clarify aspects of our condition that have never been addressed, and help break down taboos stemming from outdated cultural legacies.
- After so many years without answers, I finally found the right kind of attention.
- The hope of finding strategies to improve the lives of people with the Fragile X premutation.
- I hope that research always moves forward, and I thank those who fight for it!!!!
- First of all, I can say that I did not feel judged, and I felt at ease with both researchers.
  I’m glad there are people in the world like these doctors who conduct research and screenings to be of help in the future. Thank you.

**Question 11:**

- Extend the research to family members
- Participating made me feel cared for, but also highlighted a sense of difference in me. Perhaps a more specific initial support would be helpful
- Theorize and plan a rehabilitative path, both physical and psychological
- Continue
- Less mechanical, more dialogue
- Group meetings might be helpful, guided by specialized professionals, for people with this condition
- Also talk about the physical health of healthy carrier mothers
- Beyond answering questions and doing tests, it would be good to include some moments of open dialogue. I think it would be useful to repeat this over time, as even just “the last 15 days” can bring changes :)
- Psychological support is useful
- What should a healthy carrier do at the first signs to improve their condition?
- Prevent early menopause and Monitor general health status
